# Discoidin domain receptor 2 is an important modulator of BMP signaling during heterotopic bone formation

**DOI:** 10.1038/s41413-024-00391-z

**Published:** 2025-01-02

**Authors:** Fashuai Wu, Chunxi Ge, Haichun Pan, Yuanyuan Han, Yuji Mishina, Vesa Kaartinen, Renny T. Franceschi

**Affiliations:** 1 Department of Periodontics & Oral Medicine, University of Michigan School of Dentistry, Ann Arbor, MI USA; 2 Department of Orthopaedics, Union Hospital, Tongji Medical College, Huazhong University of Science and Technology, Wuhan, China; 3 Department of Biologic and Materials Sciences, University of Michigan School of Dentistry, Ann Arbor, MI USA

**Keywords:** Bone, Pathogenesis

## Abstract

Bone morphogenetic proteins are essential for bone regeneration/fracture healing but can also induce heterotopic ossification (HO). Understanding accessory factors modulating BMP signaling would provide both a means of enhancing BMP-dependent regeneration while preventing HO. This study focuses on the ability of the collagen receptor, discoidin domain receptor 2 (DDR2), to regulate BMP activity. As will be shown, induction of bone formation by subcutaneous BMP2 implants is severely compromised in *Ddr2*-deficient mice. In addition, *Ddr2* deficiency attenuates HO in mice expressing the ACVR1 mutation associated with human fibrodysplasia ossificans progressiva. In cells migrating into BMP2 implants, DDR2 is co-expressed with GLI1, a skeletal stem cell marker, and DDR2/GLI1-positive cells participate in BMP2-induced bone formation where they contribute to chondrogenic and osteogenic lineages. Consistent with this distribution, conditional knockout of *Ddr2* in *Gli1-*expressing cells inhibited bone formation to the same extent seen in globally *Ddr2*-deficient animals. This response was explained by selective inhibition of *Gli1*^+^ cell proliferation without changes in apoptosis. The basis for this DDR2 requirement was explored further using bone marrow stromal cells. Although *Ddr2* deficiency inhibited BMP2-dependent chondrocyte and osteoblast differentiation and in vivo, bone formation, early BMP responses including SMAD phosphorylation remained largely intact. Instead, *Ddr2* deficiency reduced the nuclear/cytoplasmic ratio of the Hippo pathway intermediates, YAP and TAZ. This suggests that DDR2 regulates Hippo pathway-mediated responses to the collagen matrix, which subsequently affect BMP responsiveness. In summary, DDR2 is an important modulator of BMP signaling and a potential therapeutic target both for enhancing regeneration and treating HO.

## Introduction

Bone loss caused by trauma, fractures, tumor resection, or congenital disease is a major cause of disability and human suffering. Several regenerative approaches have been developed to repair bone loss including treatment with bone morphogenetic proteins (BMP). BMPs are a natural component of the bone extracellular matrix (ECM) with well-defined roles in bone development and fracture healing (for reviews^1,2^). In the clinic, BMP2 and 7 have been successfully used to repair nonunion fractures and in spinal fusion procedures. However, there are major concerns related to the use of these molecules including the high doses that must be administered for successful regeneration, and related toxicity and possible oncogenesis^3–5^. On the other hand, abnormal BMP pathway activation is associated with heterotopic ossification. This can be caused either by severe trauma or mutations in the BMP type I receptor, ACVR1, as is seen in fibrodysplasia ossificans progressiva^6,7^. To address these issues, a more complete understanding is needed of accessory factors able to modulate BMP activity.

It has been known for many years that skeletal progenitor cells (SPCs) must synthesize a collagenous extracellular matrix (ECM) to fully respond to BMPs^8,9^. In addition, ECM mechanical properties such as stiffness further enhance BMP responsiveness (for reviews^10,11^). Understanding the basis for this ECM requirement could provide new strategies for both enhancing BMP activity in regeneration and reducing deleterious effects of pathological BMP pathway activation. Bone cells contain two different types of collagen receptors that mediate the response to ECM, the collagen-binding β1 integrins (e.g., α1β1, α2β1, α10β1, α11β1 integrins) and the discoidin domain receptors (DDR1 and DDR2). To date, only integrins have been implicated in BMP responsiveness. Specifically, blocking antibodies to α1 and α2 integrin subunits inhibits the ability of BMP2 to stimulate osteoblast differentiation of skeletal progenitor cells (SPCs)^9,12–14^. Also, BMP2-dependent osteoblast differentiation is defective in calvarial cells from β1 integrin-deficient mice^15^. In addition, synthetic triple-helical peptides containing the integrin-binding site from fibrillar collagens (sequence: GFOGER), when coupled to synthetic hydrogels, increase the localization of skeletal progenitor cells to the regeneration site and enhance in vivo BMP responsiveness^16^.

The discoidin domain receptors are a second less studied class of collagen receptors having important functions in bone development and regeneration (for review^17^). Unlike integrins, DDRs contain intrinsic tyrosine kinase activity that is stimulated by binding to a specific sequence in fibrillar collagens (GVMGFO) distinct from the integrin-binding sequence. While both DDR1 and DDR2 have reported activities on bone, *Ddr2* is preferentially expressed in the skeleton and the consequences of bone-selective *Ddr2* inactivation are more dramatic than for *Ddr1*. Specifically, global knockout or conditional inactivation of *Ddr2* in GLI1-positive SPCs or chondrocytes inhibits the growth of the appendicular and cranial skeletons with preferential effects on cartilage proliferation of growth plates, and cranial synchondroses, cranial suture fusion, osteoblast differentiation, and bone formation^18–20^. In contrast, conditional knockout in mature osteoblasts did not affect skeletal development, suggesting that DDR2 functions predominantly in earlier stages of cartilage and bone formation. *Ddr2*-deficient mice are also unable to heal calvarial defects or tibial fractures, consistent with DDR2 having important functions in bone regeneration, a process in part dependent on BMP signaling^21,22^. Further evidence for DDR2 functioning in progenitor populations comes from lineage tracing studies showing DDR2 is expressed in cranial suture and long bone growth plate cell populations that can differentiate into chondrocytes, osteoblasts, and osteocytes during bone development^19,20^. These DDR2^+^ cells exhibit considerable overlap with the SPC marker, GLI1. In more detailed studies, a DDR2^+^ SPC population was recently identified in cranial sutures that are distinct from CTSK^+^ SPCs and can form bone via an endochondral process. This stem cell population may be responsible for the abnormal bone formation seen in certain forms of craniosynostosis^23^. Human *DDR2* mutations cause the rare autosomal recessive disorder, spondylo-meta-epiphyseal dysplasia with short limbs and abnormal calcifications (SMED SL-AC), characterized by severe defects in skeletal and craniofacial growth, tooth abnormalities, and low bone density, confirming that DDR2 is also required for normal human skeletal development^24^. Consistent with DDR2-collagen interactions being critical for its actions in bone, the growth of skeletal progenitor cells on synthetic triple-helical peptides containing the DDR2 binding sequence from fibrillar collagens (GVMGFO) stimulates osteoblast differentiation, a response that is synergistically stimulated in the presence of integrin-binding peptides^25^.

Given the important functions for DDR2 in skeletal development and regeneration, processes known to involve BMP activity, we considered it important to determine if DDR2 is also required for BMP-induced bone formation using both a subcutaneous implant bone formation model as well as a murine model of FOP. In addition, the mechanistic basis for DDR2 responsiveness was examined using wild type and *Ddr2*-deficient bone marrow stromal cells.

## Results

### Participation of *Ddr2* in BMP2-dependent ectopic bone formation

**Fig. 1:**
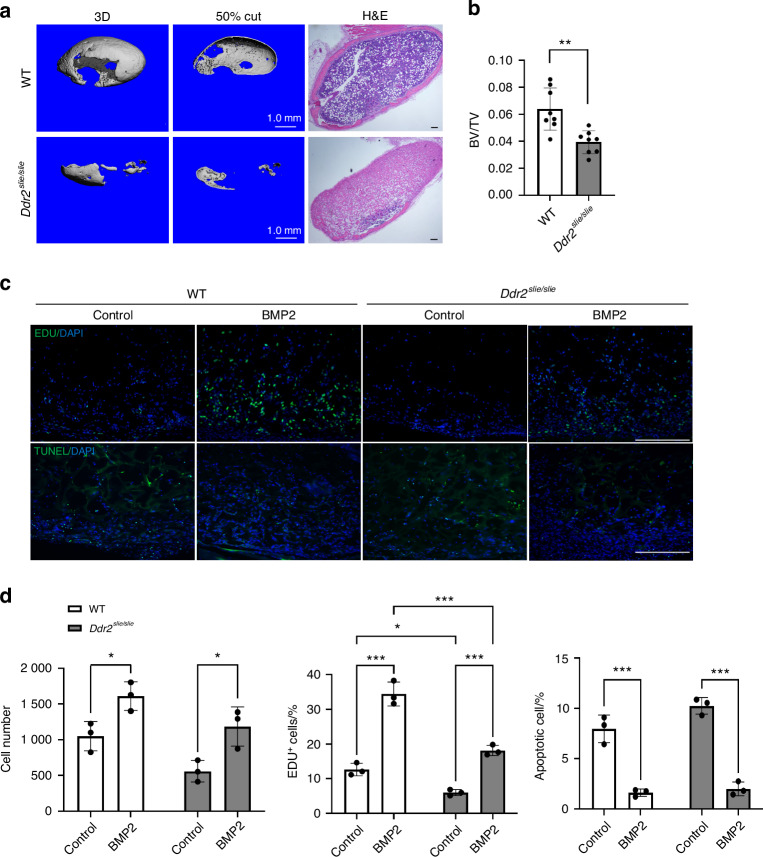
Requirement for *Ddr2* in BMP2-induced bone formation. Control or BMP2 implants were subcutaneously placed in wild type and *Ddr2*^*slie/slie*^ mice as described in “Methods” section and harvested after 4 weeks for measurement of bone formation (**a**, **b**) or after 1 week for measurement of proliferation and apoptosis (**c**, **d**). **a** 3D surface and internal (50% cut) rendering of μCT scans and histology images (right 2 panels) of ectopic bone formation in BMP2 implants from wild type and *Ddr2*^*slie/slie*^ mice. Scale bar: 200 μm. **b** Quantification of μCT images. **c** Fluorescence images of EdU labeling (proliferation, top row) and TUNEL staining (apoptosis, bottom row) of control or BMP2 implants placed in wild type or *Ddr2*^*slie/slie*^ mice. Total cell nuclei were also stained with DAPI. Scale bar: 200 μm. **d** Measurement of total cell number from DAPI staining (L), proliferation (percentage of total DAPI^+^ cells that were EdU^+^, middle), and apoptosis (percentage of DAPI^+^ cells that were TUNEL^+^, R). Statistics: Unpaired *t*-test (**b**) or two-way ANOVA (**d**), *P < 0.05, **P < 0.01, ***P < 0.001

Mice lacking BMP2 in early osteoprogenitor cells do not heal bone fractures^26^. Consistent with this essential function in fracture healing, BMP2 has been successfully used in the clinic to heal nonunion fractures and promote spinal fusions^3^. Given the important functions of this BMP in bone regeneration, our studies selectively examined the consequences of *Ddr2* deficiency on BMP2-induced osteogenesis using an ectopic bone formation assay. In this system, which is a model for both BMP-induced osteogenesis and heterotopic ossification (HO), bone forms through an endochondral process (for review^6^). In our experiments, recombinant BMP2 was adsorbed to gelatin sponges and subcutaneously implanted into 10–12-week-old wild type or *Ddr2*-deficient (*Ddr2*^*slie/slie*^) mice. After 4 weeks, implants were recovered and examined for bone formation using μCT and histology. For bone formation to occur, cells must migrate into BMP2 implants from adjacent connectives where, in response to BMP2, they proliferate and differentiate into cartilage and bone. In wild-type mice, BMP2 implants stimulated robust formation of ectopic bone containing a cortical shell, internal trabeculae, and a marrow cavity. In contrast, bone formation was severely reduced in *Ddr2*-deficient animals (Fig. 1a, b). Only small cortical and trabecular regions were detected, and internal portions of the implant contained residual gelatin matrix without visible marrow. Implants without added BMP2, regardless of the *Ddr2* status of the host animal, did not form bone although they still contained cells (detected by DAPI staining) that had migrated into gelatin sponges during the in vivo incubation period.

To begin investigating the cellular mechanism(s) responsible for the defective BMP2 response in *Ddr2*^*slie/slie*^ mice, we assessed proliferation and apoptosis. For proliferation measurements, mice were intraperitoneally injected with EdU 6 days after BMP2 implant placement. Implants were subsequently harvested and analyzed for the percentage of EdU-positive cells (Fig. 1c). BMP2 stimulated EdU incorporation in both wild type and *Ddr2*^*slie/slie*^ hosts. However, levels of EdU^+^ cells were significantly reduced in *Ddr2*-deficient implants regardless of BMP status (53 percent inhibition in controls and 42 percent inhibition with BMP2). Cell apoptosis was also measured in the same study using a fluorescent TUNEL assay (Fig. 1d). In both wild type and *Ddr2*^*slie/slie*^ hosts, BMP2 strongly inhibited apoptosis (approximately 80 percent inhibition), but apoptosis was not affected by *Ddr2* deficiency.

### DDR2-positive cells and their progeny participate in all stages of BMP2-dependent bone formation

**Fig. 2:**
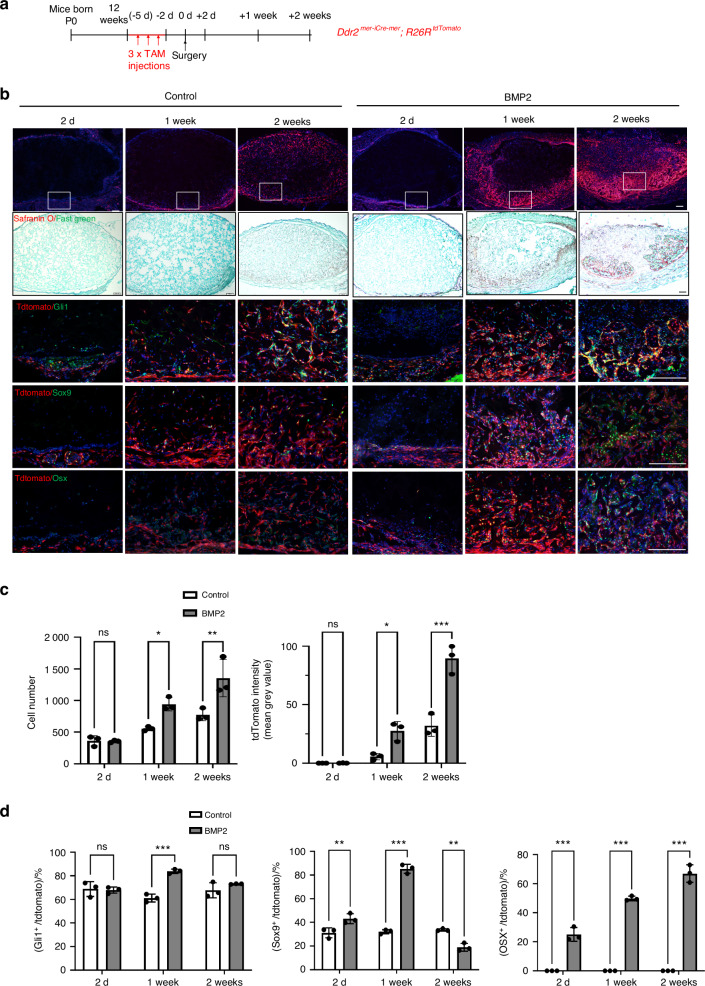
Contribution of *Ddr2* lineage cells to bone formation. **a** Experiment protocol. *Ddr2*^*mer-iCre-mer*^*; R26R*^*tdTomato*^ mice were given 3 daily tamoxifen injections followed by placement of control or BMP2 implants. Samples were harvested at the indicated times and frozen sections were prepared for immunofluorescence analysis. **b** Contribution of *Ddr2*-expressing cells and their progeny (tdTomato^+^ cells) to BMP2-induced osteogenesis and colocalization with GLI1, SOX9, and OSX. Regions of cartilage formation were also identified using Safranin O/Fast Green staining (2nd row). **c** Quantification of total cells (DAPI^+^, L) and tdTomato intensity (R) in control or BMP2 implants. **d** Quantification of tdTomato^+^ cell colocalization with GLI1, SOX9 and OSX. Statistics: two-way ANOVA. *P < 0.05, **P < 0.01, ***P < 0.001, ns not significant. Scale bar: 200 μm

The subcutaneous tissue where BMP2 was implanted in the above study contains loose mesoderm-derived connective tissue that links skin to muscle. Within this connective tissue and adjacent muscle interstitium are mesenchyme-derived progenitor cells capable for differentiating into bone when exposed to BMPs or other stimuli such as trauma^6^. Different markers have been used to characterize these progenitor cell populations including GLI1, TIE1, PDGFRα, and PRX1^27–30^. The Hedgehog pathway intermediated, GLI1, is of particular interest since, as pointed out in the Introduction, we previously showed that *Ddr2* functions in *Gli1*-expressing cells to control skeletal development^19,20^. To identify the *Ddr2*-expressing cells participating in the BMP response and determine the relationship between these cells and the different skeletal cell types involved in endochondral bone formation, control or BMP2 implants were placed in tamoxifen-treated *Ddr2*^*mer-icre-mer*^*; R26R*^*tdtomato*^ mouse hosts (Fig. 2). These animals contain tamoxifen (TAM)-activated Cre recombinase under the control of the *Ddr2* gene. After Cre-mediated recombination, mice express a tdTomato fluorescent protein in *Ddr2*-expressing cells and their progeny, thereby allowing us to trace the lineage of these cells during bone formation. Twelve-week-old *Ddr2*^*mer-icre-mer*^*; R26R*^*tdtomato*^ mice were given 3 daily i.p. TAM injections and, 2 days after the last injection, mice were implanted with control or BMP2-containing gelatin scaffolds. Implants were subsequently harvested after 2 days, 1 week, or 2 weeks (Fig. 2a). Cryosections were then analyzed by fluorescence microscopy and immunofluorescence (Fig. 2b) to measure the accumulation of total (DAPI^+^) and tdTomato^**+**^ cells in implants (Fig. 2c) and colocalization of tdTomato^+^ cells with different cell populations (Fig. 2d). In both control and BMP2 implants, total DAPI^+^ and tdTomato^+^ cells were initially present in connective tissue adjacent to implants and on the implant surface (day 2). Over time, both cell populations accumulated in implants. However, tdTomato^+^ cells greatly increased in BMP2-treated samples and preferentially accumulated in regions undergoing cartilage formation as measured by Safranin O/Fast Green staining in adjacent sections (Fig. 2b). BMP2 also increased total DAPI^+^ cells, but to a lesser extent than see with tdTomato^+^ cells (Fig. 2c). For example, at 2 weeks, BMP2 increased total DAPI^+^ cells by 75 percent while increasing tdTomato^+^ cells by 290 percent. In contrast, in the absence of BMP2, while tdTomato-positive and negative cells accumulated in implants over time, they were more randomly distributed throughout implants. These results suggest *Ddr2*-expressing cells are preferentially responsive to BMP2 and their progeny are major contributors to subsequent BMP2-induced bone formation. Further evidence for this concept was obtained when the colocalization of tdTomato^+^ cells with markers for different cell populations was examined by immunofluorescence (Fig. 2b, quantified in d). At all times examined, *Ddr2*-expressing cells and their progeny showed a high degree of colocalization with the SPC marker, GLI1 (70-85 percent of tdTomato^+^ cells were GLI1^+^), and this colocalization was minimally affected by BMP2. In contrast, colocalization with SOX9, a chondrocyte/chondroprogenitor cell marker^31^, remained low at all time points in the absence of BMP2 (approx. 30 percent colocalization) but was preferentially stimulated by BMP2 beginning at day 2, peaking after 1 week (85 percent colocalization) and then declining to below control levels at 2 weeks. Lastly, in the absence of BMP2, very few cells stained positive for the osteoblast/preosteoblast marker, OSX (SP7), while in BMP2-treated mice approximately 25 percent of tdTomato^+^ cells were OSX^+^ at 2 days and this value increased to 50 percent at 1 week and then to 70 percent after 2 weeks in parallel with the increase in bone formation. These results are what would be expected if *Ddr2* was expressed in a GLI1^+^ SPC population that first formed SOX9^+^ chondrocytes and then OSX^+^ osteoblasts as BMP-induced endochondral bone formation proceeded.

### *Ddr2* functions in GLI1-positive cells to stimulate BMP2-induced bone formation

**Fig. 3:**
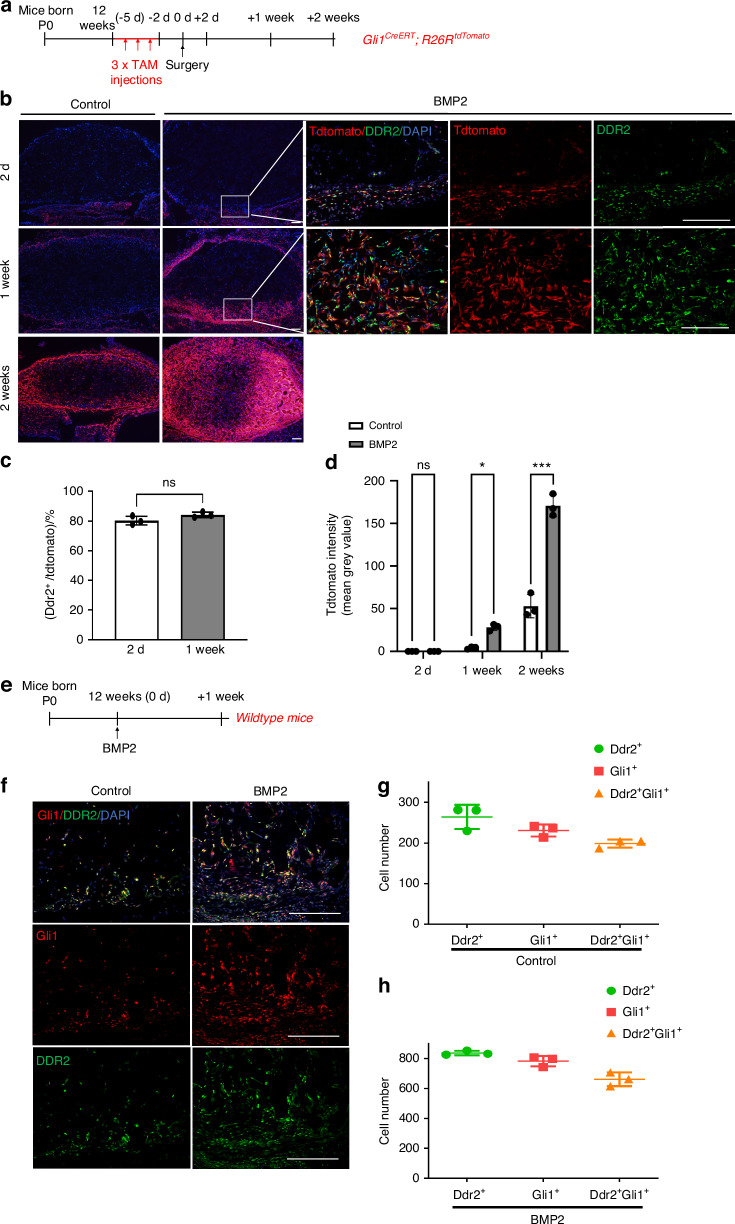
Distribution of *Gli1*-expressing cells during bone formation and colocalization with DDR2. **a** Experiment protocol. *Gli1*^*CreERT*^*; R26R*^*tdTomato*^ mice were subjected to the same induction protocol described Fig. 2. **b** Distribution of *Gli1*-expressing cells in control and BMP2 implants. For 2-day and 1 week BMP2 implants, colocalization of tdTomato (red) with DDR2 (green) was measured by IF and is quantified in (**c**). **d** Quantification of tdTomato intensity over time. **e** Experimental protocol for GLI1/DDR2 IF colocalization study. Control or BMP2 implants were placed in wild-type mice and harvested after 1 week. **f** IF colocalization of GLI1 (red) and DDR2 (green) in control and BMP2 implants. **g**, **h** Quantification of colocalization results. DDR2^+^, GLI1^+^, and DDR2^+^/GLI1^+^ cell numbers are indicated in control (**g**) and BMP2 implants (**h**). Statistics: Unpaired *t*-test, *P < 0.05, ***P < 0.001, ns not significant. Scale bar: 200 μm

GLI1^+^ progenitor cells were previously identified in subcutaneous connective tissue and shown to be major BMP targets during injury-induced heterotopic ossification^27,30^. The high colocalization of tdTomato^+^ cells with GLI1 in *Ddr2*^*mer-icre-mer*^*; R26R*^*tdtomato*^ mice (Fig. 2) suggests that DDR2 may largely function in this progenitor population. To determine if a DDR2^+^ GLI1^+^ progenitor population participates in BMP2-induced bone formation, *Gli1-Cre*^*ERT*^; *R26R*^*tdTomato*^ mice were injected with TAM under the same conditions used with *Ddr2*^*mer-iCre-mer*^; *R26R*^*tdTomato*^ mice and analyzed for tdTomato distribution at various times up to two weeks after implantation with control or BMP2-containing scaffolds (Fig. 3). In the presence of BMP2, tdTomato labeling was initially detected in the subcutaneous connective tissue surrounding the implants with few tdTomato^+^ cells observed inside implants. At later time points, tdTomato^+^ cells expanded into the implants where they were preferentially associated with regions of bone and cartilage formation. When compared with control implants, there were much more tdTomato^+^ cells in implants containing BMP2 (Fig. 3b, d). This distribution was like that seen in *Ddr2*^*mer-icre-mer*^*; R26R*^*tdtomato*^ mice, suggesting that there is substantial overlap between *Ddr2* and *Gli1*-expressing cells and their progeny. Furthermore, immunofluorescence analysis with a DDR2 antibody showed that approximately 80 percent of tdTomato^+^ cells from *Gli1-Cre*^*ERT*^; *R26R*^*tdTomato*^ mice were stained with DDR2 antibody in BMP2 implants (Fig. 3c). Lastly, direct immunofluorescence analysis with anti-DDR2 and anti-GLI1 antibodies showed that approximately 85 percent of GLI1^+^ cells in implants also contained DDR2 regardless of whether BMP2 was present (Fig. 3f–h). Thus, GLI1^+^ cells are major contributors to BMP2-dependent bone formation and most GLI1^+^ cells also contain DDR2.

**Fig. 4:**
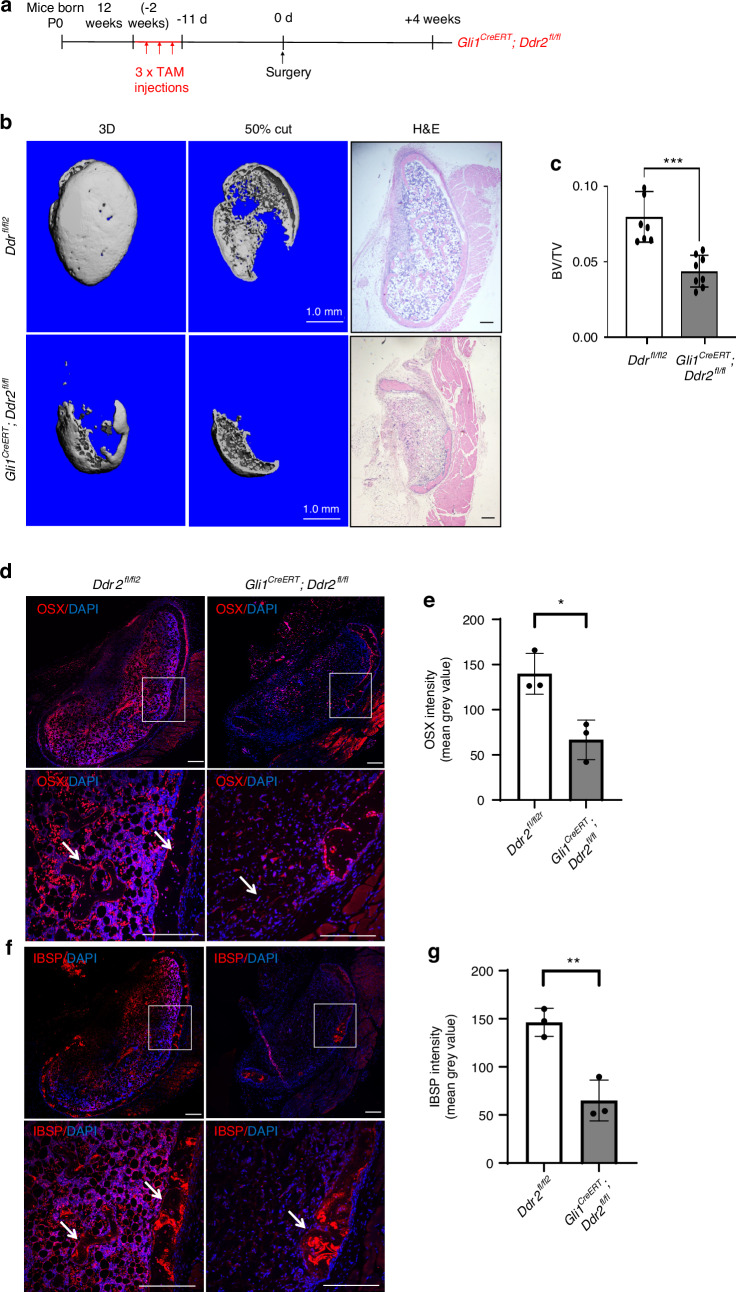
Inactivation of *Ddr2* in *Gli1*-expressing cells inhibits BMP2-induced bone formation. **a** Experimental protocol for *Ddr2*^*fl/fl*^ *and Gli1*^*CreERT*^*; Ddr2*^*fl/fl*^ mice. **b** 3D surface and internal (50% cut) rendering of μCT scans and histology images (R panel) of ectopic bone formation in BMP2 implants from tamoxifen-treated *Ddr2*^*fl/fl*^ *and Gli1*^*CreERT*^; *Ddr2*^*fl/fl*^ mice. Scale bar: 1.0 mm for μCT, 200 μm for histology. **c** Quantification of μCT images. **d**, **e** IF staining and quantification for OSX. **f**, **g** IF staining and quantification for IBSP. Statistics: unpaired *t*-test, *P < 0.05, **P < 0.01, ***P < 0.001 Low and high power images are shown. Scale bar: 200 μm

To assess the cell-autonomous function of DDR2 in GLI1^+^ SPCs, we employed a conditional deletion approach using *Gli1-Cre*^*ERT*^; *Ddr2*^*fl/fl*^ mice (Fig. 4). These mice and controls (*Ddr2*^*fl/fl*^) were injected with TAM and implanted with control or BMP2 scaffolds. After TAM treatment, *Ddr2* was efficiently inactivated by *Gli1-Cre*^*ERT*^ (Fig. S1). Four weeks after BMP2 implantation, ossicles were harvested and analyzed by microCT, histology, and immunofluorescence microscopy. BMP2 implants in *Gli1-Cre*^*ERT*^; *Ddr2*^*fl/f*^ mice exhibited a large reduction in BV/TV compared with littermate controls. This phenotype was comparable to that seen in Ddr2^slie/slie^ mice where *Ddr2* was inactivated in all cells (approx. 50 percent inhibition of bone formation as measured by μCT). Furthermore, decreased IF staining for the osteoblast/preosteoblast markers, OSX and IBSP, was observed when knockout and control mice were compared (Fig. 4d–g).

**Fig. 5:**
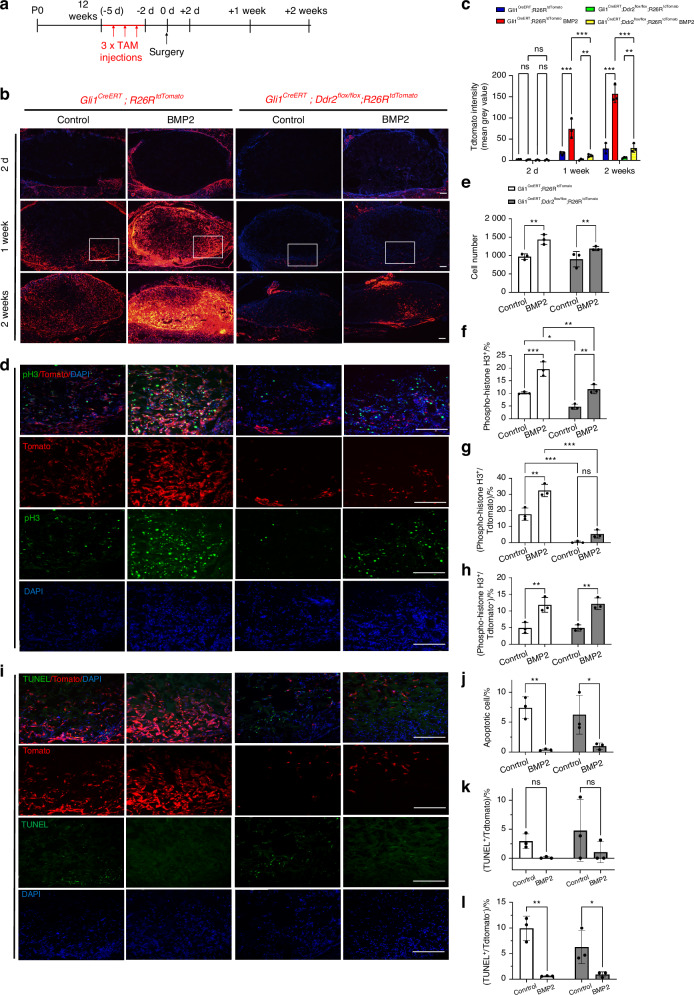
Selective effects of *Ddr2* inactivation in *Gli1*-expressing cells. **a** Experimental protocol. After tamoxifen treatment, *Gli1*^*CreERT*^*; R26R*^*tdTomato*^ and *Gli1*^*CreERT*^*; Ddr2*^*flox/flox*^*; R26R*^*tdTomato*^ mice received control or BMP2 implants that were harvested after 2 days, 1 week or 2 weeks for measurement of tdTomato fluorescence (**b**, **c**). One-week samples were also analyzed for cell number (DAPI staining, **d**, **e**), proliferation using IF staining with a phospho-histone H3 (pH3) antibody (**d**, **f**–**h**), or for apoptosis using a fluorescence TUNEL assay (**i**–**l**). Key: **c** *Gli1*^*CreERT*^*; R26R*^*tdTomato*^ (blue bars), *Gli1*^*CreERT*^*; R26R*^*tdTomato*^ + BMP2 (red bars), *Gli1*^*CreERT*^*; Ddr2*^*flox/flox*^*; R26R*^*tdTomato*^ (green bars), *Gli1*^*CreERT*^*; Ddr2*^*flox/flox*^*; R26R*^*tdTomato*^ + BMP2 (yellow bars). **d**–**l** *Gli1*^*CreERT*^*; R26R*^*tdTomato*^ (open bars), *Gli1*^*CreERT*^*; Ddr2*^*flox/flox*^*;R26R*^*tdTomato*^ (closed bars). Statistics: two-way ANOVA. *P < 0.05, **P < 0.01, ***P < 0.001, ns not significant. Scale bar: 200 μm

To determine which stages of endochondral ossification are disrupted by Ddr2 inactivation and gain further insight into selective functions of DDR2 in GLI1^+^ cells, *Gli1*^*CreERT*^*; R26R*^*tdTomato*^ and *Gli1*^*CreERT*^*; Ddr2*^*fl/fl*^*; R26R*^*tdTomato*^ mice were treated with TAM, implanted with control and BMP2 scaffolds and examined after 2 days, 1 week or 2 weeks (Fig. 5, Fig. S2). This approach allowed us to examine the consequences of *Ddr2* deficiency specifically in *Gli1*-expressing cells and their progeny over time. DDR2 protein levels as well as the stage/s of BMP-induced endochondral ossification disrupted by *Ddr2* inactivation were examined by evaluating histological sections from 1- and 2-week samples (Fig. S2). Samples were evaluated for DDR2 protein (panel d), cartilage formation (Safranin O/Fast Green staining, SOX9-panels a,b) and early osteoblast marker induction (OSX, panel c). *Gli1*^*CreERT*^-mediated *Ddr2* inactivation greatly reduced DDR2 immunofluorescence at both time points (approx. 90 percent inhibition), confirming that recombination induced by this Cre strongly inhibited DDR2 protein levels in implants. Loss of DDR2 also partially inhibited cartilage formation/marker expression at 1 week and subsequent osteoblast formation at 2 weeks. This confirms that loss of DDR2 inhibits both early and late stages of BMP-induced endochondral bone formation.

In agreement with results shown in Fig. 3, tdTomato^+^ cells in *Gli1-Cre*^*ERT*^*; R26R*^*tdTomato*^ mice increased in both control and BMP2 implants with the rate of cell accumulation being much greater in the presence of BMP2 (Fig. 5b, c). In contrast, *Ddr2* inactivation in Gli1-expressing cells (*Gli1-Cre*^*ERT*^; *Ddr2*^*fl/fl*^*; R26R*^*tdTomato*^ mice) dramatically reduced the time-dependent increase in tdTomato^+^ cells. This result could not be explained by *Ddr2*-inactivation reducing the efficiency of recombination at the *R26R*^*tdTomato*^ locus since the initial (day 2) levels of tdTomato^+^ cells were similar in all 4 groups (*Gli1-Cre*^*ERT*^*; R26R*^*tdTomato*^ control, *Gli1-Cre*^*ERT*^*; R26R*^*tdTomato*^ BMP2, *Gli1-Cre*^*ERT*^*;Ddr2*^*fl/fl*^*; R26R*^*tdTomato*^ control, *Gli1-Cre*^*ERT*^*;Ddr2*^*fl/fl*^*; R26R*^*tdTomato*^ BMP2). In addition, levels of tdTomato^+^ cells in tibial growth plates from all 4 groups of mice were similar as would be expected if recombination was not affected by *Ddr2* status (Fig. S3). Effects of *Ddr2* inactivation, as expected, were specific to tdTomato^+^ cells. For example, for the 1 week BMP2 group, *Ddr2* inactivation inhibited tdTomato fluorescence by approx. 88 percent while total cells (DAPI^+^) were not significantly reduced (Fig. 5c vs. e). This reflects the fact that only a small fraction of cells entering implants are tdTomato-positive and possible expansion of non-tdTomato^+^ cells.

To delve more deeply into the basis for the reduction in GLI1-derived tdTomato^+^ cells in *Ddr2*-deficient mice, cell proliferation was measured in total (DAPI^+^) and tdTomato^+^ cells using staining with a histone H3 Ser10-P (phospho-Histone H3) antibody that is specific to mitotic cells^32^. Consistent with results shown in Fig. 1, BMP2 stimulated proliferation in total DAPI^+^ cells and this response was modestly inhibited by *Ddr2* inactivation (Fig. 5f). However, *Ddr2* inactivation had a much stronger inhibitory effect when phospho-Histone H3 staining was selectively examined in GLI1-derived tdTomato^+^ cells (Fig. 5g). As further evidence of the specificity of *Ddr2* inactivation, when phospho-Histone H3 staining was examined in tdTomato-negative cells, no differences were seen when *Gli1-Cre*^*ERT*^*; R26R*^*tdTomato*^ and *Gli1-Cre*^*ERT*^*; Ddr2*^*fl/fl*^*; R26R*^*tdTomato*^ mice were compared (Fig. 5h). Apoptosis was also examined in tdTomato-positive and negative cells using a fluorescence TUNEL assay (Fig. 5i–l). In agreement with the results shown in Fig. 1, BMP2 treatment reduced overall levels of apoptosis. However, levels were not affected by *Ddr2* inactivation, and no significant differences were seen when tdTomato^+^ or tdTomato^-^ cells were compared. As shown by these experiments, loss of DDR2 in GLI1^+^ cells and their progeny selectively inhibits proliferation without affecting apoptosis leading to fewer cells being available to form new cartilage and bone after BMP2 stimulation. In contrast, BMP2-dependent inhibition of apoptosis was not affected by *Ddr2* status.

### DDR2-inactivation reduces heterotopic ossification in a mouse FOP model

**Fig. 6:**
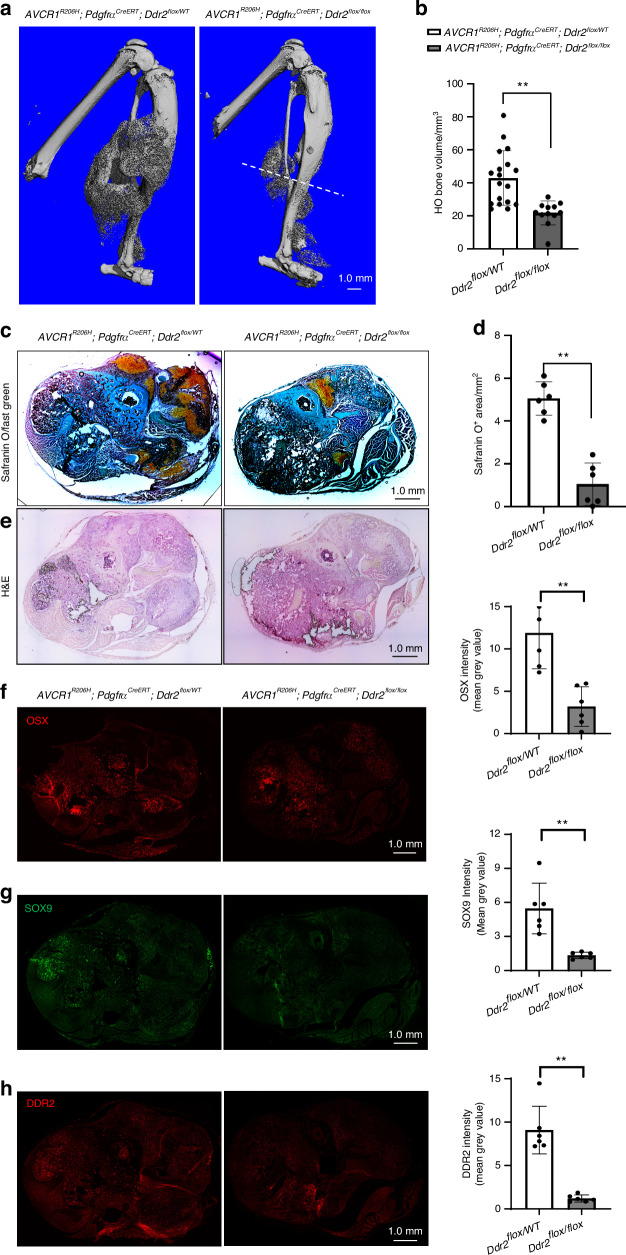
*Ddr2* inactivation reduces the HO associated with a FOP mutation in *Acvr1*. *Pdgfra*^*CreER*^*; Acvr1*^*[R206H]FlEx/+*^*; Ddr2*^*fl/+*^ and *Pdgfra*^*CreER*^*; Acvr1*^*[R206H]FlEx/+*^*; Ddr2*^*fl/fl*^ mice were generated and subjected to the HO induction protocol described in “Methods” section. Legs were harvested after 2 weeks for analysis. **a**, **b** 3D μCT renderings and quantification of HO bone volume. The large HO volume in *Pdgfra*^*CreER*^*; Acvr1*^*[R206H]FlEx/+*^*; Ddr2*^*fl/+*^ mice (*Ddr2*^*flox/+*^) was significantly reduced in *Pdgfra*^*CreER*^*; Acvr1*^*[R206H]FlEx/+*^*; Ddr2*^*fl/fl*^ mice (*Ddr2*^*fl/fl*^). The dashed line in (**a**) shows plane of sections used for histology and immunofluorescence analysis (**c**–**h**). **c**, **d** Safranin O/Fast Green staining and quantitation of Safranin O area. **e** H & E staining. **f**–**h** Immunofluorescence staining and quantification of OSX, SOX9, and DDR2. Statistics: unpaired *t*-test, **P < 0.01

Fibrodysplasia ossificans progressiva (FOP), a rare genetic disorder associated with progressive soft tissue ossification, is caused by a conserved (R206H) substitution in the BMP type I receptor, ACVR1, allowing it to be abnormally stimulated by activin A, which normally suppresses BMP stimulation of the wild-type receptor^33^. FOP is, therefore, an example of abnormal activation of BMP signaling leading to ectopic endochondral bone formation. In view of the above results showing the involvement of DDR2 in BMP2-induced ossification, we considered it important to also evaluate its possible role in FOP. To do this, we examined the consequences of *Ddr2* inactivation in a widely accepted model of FOP using *Pdgfra*^*CreERT*^*; Acvr1*^*[R206H]FlEx/+*^ mice. In this model, Cre-mediated recombination inverts the R206H-encoding exon into the sense strand while also deleting the wild-type exon leading to specific expression of the mutant allele in *Pdgfra*-expressing cells^33^. Consistency of the HO response is facilitated by subsequent induction of muscle tissue damage using i.m. cardiotoxin injection^34^. As was previously shown, *Pdgfra*^*CreERT*^ is expressed in a mesenchyme-derived fibro/adipogenic progenitor population that is able to efficiently induce HO after *Acvr1*^*[R206H]FlEx/+*^ recombination^6,35^. For our studies, a *Ddr2*^*fl/fl*^ allele was introduced into *Pdgfra*^*CreERT*^*;Acvr1*^*[R206H]FlEx/+*^ mice to generate the following genotypes: *Pdgfra*^*CreERT*^*;Acvr1*^*[R206H]FlEx/+*^*;Ddr2*^*fl/+*^ and *Pdgfra*^*CreERT*^*;Acvr1*^*[R206H]FlEx/+*^*;Ddr2*^*fl/fl*^. After systemic tamoxifen treatment and cardiotoxin injection into the muscles of the lower hindlimb, *Pdgfra*^*CreERT*^*; Acvr1*^*[R206H]FlEx/+*^*; Ddr2*^*fl/+*^mice formed large areas of HO near the injection site as measured by μCT and histology. However, the introduction of the *Ddr2*^*fl/fl*^ allele reduced the HO volume by approximately 50 percent (Fig. 6a, b), a similar inhibitory effect to that seen in BMP2-induced HO. Strong DDR2 IF staining was detected within the HO region of *Pdgfra*^*CreERT*^*; Acvr1*^*[R206H]FlEx/+*^*; Ddr2*^*fl/+*^ mice. This staining was reduced by 90 percent in *Pdgfra*^*CreERT*^*; Acvr1*^*[R206H]FlEx/+*^*; Ddr2*^*fl/fl*^ animals indicating that the *Pdgfra*^*CreERT*^ was able to efficiently knockout *Ddr2* in cells participating in HO (Fig. 6h). *Ddr2* inactivation also reduced cartilage formation as measured by Safranin O/Fast Green staining (80 percent inhibition, Fig. 6c, d). The reduction in HO was also reflected by reductions in SOX9 and OSX immunofluorescence (Fig. 6f, g). Therefore, DDR2 is necessary not only for BMP2-induced ectopic bone formation but also for HO caused by pathological activation of BMP signaling caused by ACVR1 mutation. Control studies showed HO formation to be equivalent when *Pdgfra*^*CreERT*^*; Acvr1*^*[R206H]FlEx/+*^*; Ddr2*^*fl/+*^ and *Pdgfra*^*CreER*^*; Acvr1*^*[R206H]FlEx/+*^*; Ddr2*^*+/+*^ mice were compared. Therefore, the loss of a single *Ddr2* allele is not by itself able to inhibit FOP-induced HO (Supplementary Fig. 4).

Although the *Pdgfra*^*CreERT*^ used in FOP studies and the *Gli1-Cre*^*ERT*^ used with BMP2 implants are both expressed in mesenchyme-derived connective tissue cells, the degree of functional overlap between the cell populations targeted is currently unknown. To gain greater insight into the relative contribution of these two cell populations to HO formation, *Pdgfra*^*CreERT*^*; R26R*^*tdTomato*^ and *Pdgfra*^*CreERT*^*; Ddr2*^*fl/fl*^*; R26R*^*tdTomato*^ mice were generated and used in BMP2 implant studies for comparison with results obtained with *Gli1-Cre*^*ERT*^. Mice were treated with TAM, implanted with control or BMP2 scaffolds, and examined for tdTomato distribution after 2 days, 1 and 2 weeks, and for HO formation after 4 weeks (Figs. S5, 6). The tdTomato^+^ cells were initially in the soft connective tissue surrounding implants and gradually accumulated in implants over time. This accumulation was strongly stimulated by BMP2 (Fig. S5b). However, relative to *Gli1-Cre*^*ERT*^*; Ddr2*^*fl/fl*^*; R26R*^*tdTomato*^ mice, tdTomato^+^ fluorescence intensity in *Pdgfra*^*CreERT*^*; R26R*^*tdTomato*^ implants was quite low in both control and BMP2-treated animals (compare Fig. 3 and 5 with Supplementary Fig. 5). Nevertheless, *Ddr2* inactivation in *Pdgfra*^*CreERT*^*; Ddr2*^*fl/fl*^*; R26R*^*tdTomato*^ mice greatly reduced the number of tdTomato^+^ cells in BMP2-treated samples (80 percent inhibition after 2 weeks, Fig. S5b). It also reduced HO formation as measured by μCT analysis of 4-week samples. However, in contrast to results with *Gli1-Cre*^*ERT*^ where *Ddr2* inactivation reduced BMP-induced HO by 50 percent, HO was only reduced by approximately 18 percent in *Pdgfra*^*CreERT*^*; Ddr2*^*fl/fl*^*; R26R*^*tdTomato*^ mice (Fig. S6). It is unlikely that these differences are explained by differences in Cre activity since the recombination efficiency of the *Ddr2* allele was similar when *Gli1*^*CreER*^ and *Pdgfra*^*CreER*^ were compared (Fig. S1). These studies show that Ddr2 can function in cells derived from both GLI1^+^ and PDGFRA^+^ progenitors to promote BMP-dependent HO. However, because PDGFRA^+^ cells represent only a small fraction of the cells participating in BMP2-induced HO formation, *Ddr2* inactivation in this population reduces HO less than when it is inactivated in GLI1^+^ cells, which includes most if not all BMP2 responsive cells.

### BMP2-dependent osteogenic differentiation of BMSCs requires DDR2

**Fig. 7:**
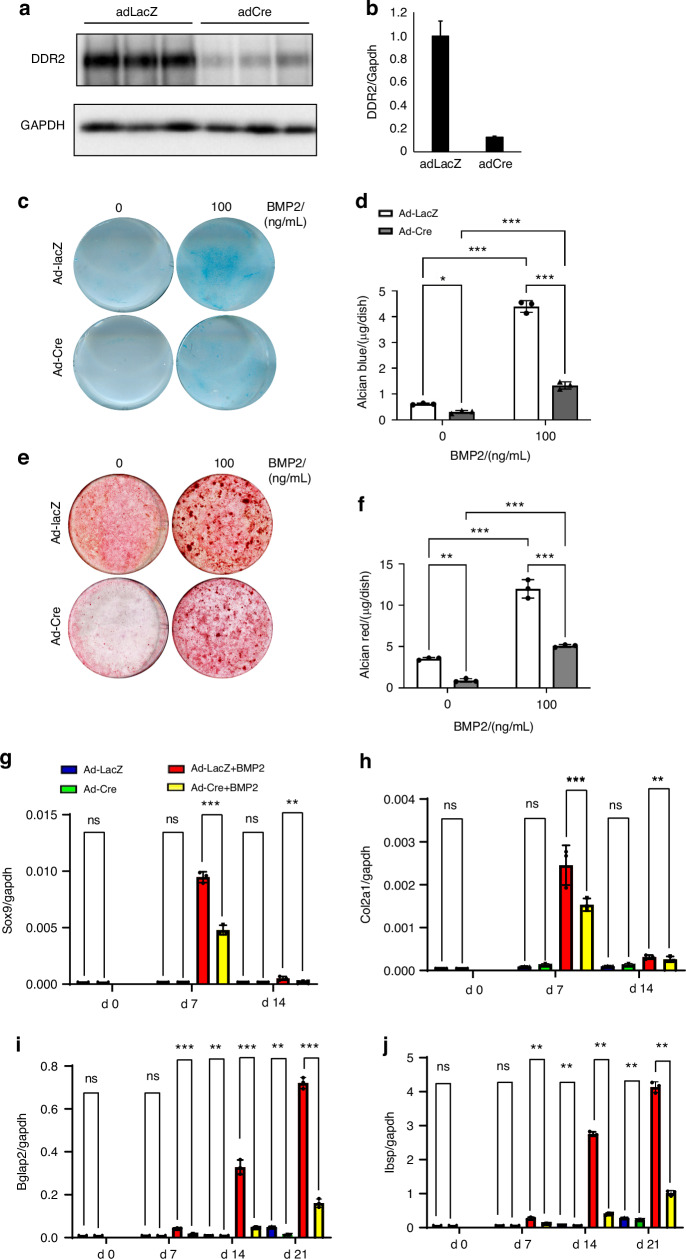
DDR2 is required for BMP2-induced chondrocyte and osteoblast differentiation of BMSCs. BMSCs were isolated from *Ddr2*^*fl/fl*^ mice, treated with control (Ad-LacZ) or Cre adenovirus (Ad-Cre), and grown in the presence or absence of BMP2 (100 ng/mL) before analysis of cartilage and bone markers as described in “Methods” section. **a**, **b** DDR2 protein levels. DDR2 protein was measured by Western blot (**a**) and quantified by densitometry after normalization to GAPDH (**b**). **c**, **d** Alcian blue staining and quantification of cartilage glycosaminoglycans measured at day 7. **e**, **f** Mineralization (Alizarin Red staining) and quantification measured at d 21. **g**, **h** Time-dependent changes in chondrocyte differentiation marker mRNAs: Sox9, Col2a1. **i**, **j** Time-dependent changes in osteogenic differentiation marker mRNAs: Bglap2 and Ibsp. Statistical analysis: 2-way ANOVA. *P < 0.05, **P < 0.01, ***P < 0.001, ns not significant

To begin investigating the effects of DDR2 on BMP signaling at the cellular level, the next series of experiments examined bone marrow stromal cells (BMSCs) in cell culture. These cells are enriched in SPCs expressing *Ddr2* and *Gli1*, have the capacity to undergo chondrogenic and osteogenic differentiation in cell culture, and form bone after in vivo implantation; they are also used extensively in bone regeneration applications^19,36^. Previous studies from our group showed that osteoblast differentiation is reduced in BMSCs from Ddr2-deficient mice^18^, but the BMP responsiveness of these cells has not been previously examined. Cells were isolated from *Ddr2*^*fl/f*^ mice, transduced with control (Ad-LacZ) or Cre-expressing adenovirus (Ad-Cre), and grown in an osteogenic medium with or without BMP2 for up to 21 days. Adeno-Cre treatment reduced DDR2 protein levels by approximately 90 percent (Fig. 7a, b). BMSCs were observed to transiently differentiate into chondrocytes after BMP2 treatment (Fig. 7c, d, g, h). This response, as measured by Alcian Blue staining and expression of chondrocyte marker mRNAs (*Sox9* and *Col2a1*) is maximally stimulated after 1 week of BMP2 treatment and returns to control levels at week 2. *Ddr2* inactivation inhibited both chondrocyte markers (Alcian Blue staining, 70 percent inhibition; chondrocyte mRNA expression, 50 percent). Significantly, *Ddr2* knockout also inhibited BMP2-induced osteoblast differentiation as assessed by Alizarin Red staining at day 21 (60 percent inhibition, Fig. 7e, f) and expression of osteoblast marker mRNAs (*Bglap2* and *Ibsp*) after 7, 14 and 21 days (Fig. 7i, j).

To evaluate whether loss of DDR2 also inhibits the osteogenic activity of BMSCs in vivo, Ad-LacZ (control) and Ad-Cre-transduced BMSCs were subcutaneously implanted into immunodeficient mice (Fig. S7). After 4 weeks, implants were harvested, and bone formation was assessed by μCT and histology. As expected, control cells formed well-developed bone containing a cortical shell, and internal regions of trabecular bone and marrow. *Ddr2* inactivation in Ad-Cre transduced cells reduced BV/TV by 75 percent when compared with Ad-lacZ-treated controls with only a few scattered μCT-positive mineralized areas remaining.

### Changes in early BMP2 signaling associated with Ddr2 deficiency

**Fig. 8:**
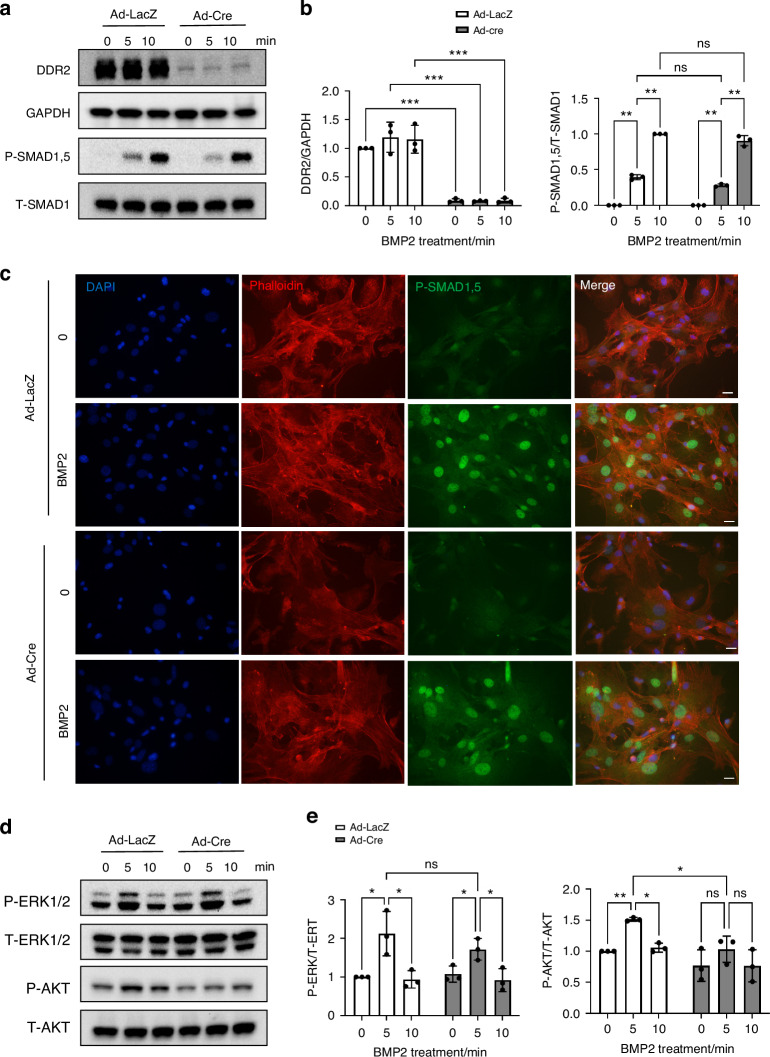
*Ddr2* deficiency is not associated with major changes in early BMP2 signaling. BMSCs from *Ddr2*^*fl/fl*^ mice were transduced with adenoviruses as described in Fig. 7 and treated with BMP2 for the times indicated before measurement of early BMP2 responses by Western blotting and immunofluorescence. **a**, **b** Western blot analysis of DDR2 and P-SMADs. P-SMAD1/5 levels were normalized to total SMAD1. **c** Subcellular distribution of P-SMADs1/5 in control and Ad-Cre-treated cells was measured by immunofluorescence microscopy 2 h after BMP2 addition. Scale bar: 20 μm. **d**, **e** Western blot analysis of ERK1/2 and AKT phosphorylation. P-ERK1/2 levels were normalized to total ERK1/2 and P-AKT levels normalized to total AKT. Statistics: Two-way ANOVA, *P < 0.05, **P < 0.01, ***P < 0.001, ns not significant

To identify DDR2-dependent aspects of BMP2 signaling, we first examined early BMP2-induced signals in wild type and *Ddr2*-deficient BMSCs. BMP‐2 initiates signaling by binding and stabilizing membrane complexes consisting of type I and type II BMP receptors. The type II receptor then phosphorylates and activates the type I receptor, recruiting and phosphorylating SMADs1/5/9 at their C‐terminal SSXS motif to initiate the canonical SMAD cascade. Phospho-SMADs1/5/9 form complexes with SMAD4 that accumulate in the nucleus where they interact with specific transcription factors to regulate gene expression. Activated BMP receptors also signal through a noncanonical pathway involving activation of TGF-β activated kinase 1 (TAK1) followed by activation of MAP kinase and PI3 kinase signaling resulting in AKT phosphorylation (for reviews^1,37^). BMSCs from *Ddr2*^*fl/fl*^ mice were transduced with control or Cre-expressing adenovirus and treated with BMP2 for increasing times (Fig. 8). Both canonical (SMAD1,5 phosphorylation and nuclear translocation) and noncanonical signals (phosphorylation of ERK1/2 and AKT) were examined. In control cells, BMP2 rapidly stimulated SMAD1/5 phosphorylation after 5 and 10 min. Surprisingly, SMAD phosphorylation was also activated to a similar extent in DDR2-deficient cells (Fig. 8a, b). Also, nuclear accumulation of SMAD1/5 as measured by immunofluorescence was normal in the presence or absence of DDR2 (Fig. 8c). Lastly, ERK1/2 and AKT phosphorylation was examined (Fig. 8d, e). In control cells, phosphorylation of both kinases increased 5 min after BMP2 addition and then returned to control levels after 10 minutes. Significant BMP2 stimulation of ERK1/2 phosphorylation was also detected in DDR2-deficient cells. However, no significant increase in AKT phosphorylation was seen. Taken together, these studies show that early canonical and noncanonical BMP signaling pathways are largely intact in the absence of DDR2 although there may be defects in AKT.

### Participation of DDR2 in Hippo signaling

Because we found early BMP signaling to be relatively independent of DDR2, we explored other possible routes through which this collagen receptor might affect BMP activity. In other systems, DDR2 functions together with the collagen-binding β1 integrins to sense and modulate the extracellular environment including the biomechanical environment. For example, in breast tumor-associated fibroblasts, DDR2 increases integrin activation in response to collagen as well as tumor stiffness by aligning type I collagen fibrils^38^.

The Hippo pathway intermediates, Yes-associated protein (YAP), and transcriptional coactivator with PDZ-binding motif (TAZ) are important mediators of the response of cells to changes in matrix-induced stiffness. These factors exist in inactive phosphorylated forms in the cytoplasm that are maintained by the Hippo kinases, LATS1 and LATS2, and in active, dephosphorylated nuclear forms that function as transcriptional coregulators together with several transcription factors including TEA domain (TEAD) factors, T-box 5 (TBX5), RUNX2, PPARγ and SMAD1 (for review^39^). Interestingly, stimulating YAP/TAZ nuclear localization by growing cells on stiff matrixes enhances BMP2-dependent osteoblast differentiation of mesenchymal cells via a pathway that is independent of changes in R-SMAD phosphorylation or nuclear translocation^10,40,41^. Although these BMP studies generally considered integrins to be the main mediators of YAP/TAZ localization via Rho/ROCK-mediated cytoskeletal stiffening, the participation of DDR2 in this process was not considered.

**Fig. 9:**
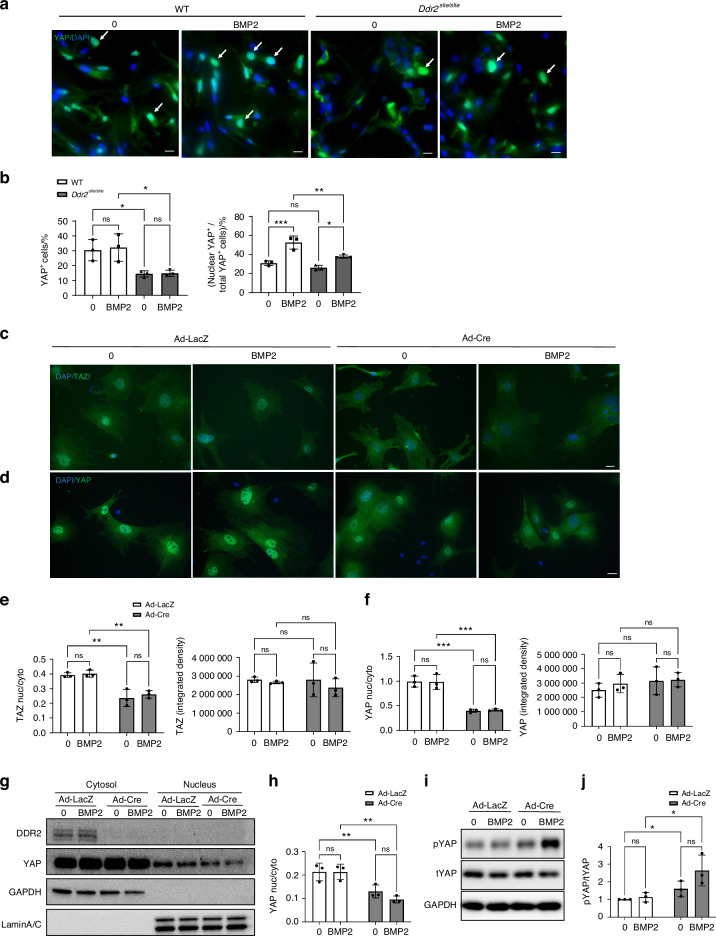
DDR2 regulates YAP/TAZ subcellular distribution. **a**, **b** In vivo YAP distribution. Control or BMP2-containing implants were placed in wild type or *Ddr2*^*slie/slie*^ mice. After 1 week, implants were examined for YAP distribution by immunofluorescence (**a**). **b** The percent of YAP-positive cells (L) and the percent of total YAP-positive cells with YAP in the nucleus (R) are indicated. **c**–**h** YAP/TAZ distribution in BMSCs. Cells from Ddr2^flox/flox^ mice were treated with control (Ad-LacZ) or Cre adenovirus (Ad-Cre) and plated on collagen-coated slides. After a 10 h attachment period, cells were treated with BMP2 for 2 h before analysis. **c**, **d** Immunofluorescence images showing TAZ and YAP nuclear/cytoplasmic distribution. **e** Quantification of nuclear/cytoplasmic distribution of TAZ fluorescence (L) and total cell TAZ fluorescence (R). **f** Quantification of nuclear/cytoplasmic distribution of YAP fluorescence (L) and total cell YAP fluorescence (R). **g**, **h** Measurement of YAP distribution by subcellular fractionation. BMSCs were treated as described above followed by separation of cells into nuclear and cytoplasmic fractions. YAP, GAPDH (cytoplasmic marker), and Lamin A/C (nuclear marker) were measured on Western blots (**g**) and quantified in (**h**). **i**, **j** Measurement of P-YAP. Whole-cell extracts were analyzed on Western blots for P-YAP and total YAP (**i**). Quantification of P-YAP/total YAP is shown in (**j**). Scale bars: 20 μm. Statistics: Two-way ANOVA, *P < 0.05, **P < 0.01, ***P < 0.001, ns not significant

To examine the possible involvement of the Hippo pathway in the DDR2 response, we initially examined YAP distribution in BMP2 implants from wildtype and *Ddr2*-deficient (*Ddr2*^*slie/slie*^) mice (Fig. 9a, b). Implants were placed as in Fig. 1 and, after 1 week, total YAP levels and nucleus/cytoplasm distribution were measured by IF. BMP2 did not affect total YAP^+^ cells regardless of *Ddr2* status. However, BMP2 increased YAP nuclear localization in wild-type mice. In contrast, in Ddr2-deficient animals, the number of YAP^+^ cells was reduced and the BMP2-dependent increase in nuclear YAP was also attenuated. To examine the relationship between *Ddr2* and Hippo signaling in greater detail, studies were conducted with BMSCs. In this case, cells from *Ddr2*^*fl/f*^ mice were transduced with control or Cre-expressing adenovirus, plated on collagen-coated surfaces, and acutely treated with BMP2 for 2 h before the distribution of YAP and TAZ was measured using immunofluorescence and subcellular fractionation(Fig. 9c–h). *Ddr2* inactivation clearly reduced the nuclear/cytoplasmic distribution of both YAP and TAZ as measured by immunofluorescence (Fig. 9c–f). However, unlike what was seen in vivo, the total YAP and TAZ signal (combined cytoplasmic and nuclear immunofluorescence) was similar in all groups indicating that results were mainly explained by changes in YAP/TAZ distribution rather than overall expression levels. Also, YAP/TAZ distribution was not affected by BMP2 treatment. The drop in nuclear YAP was confirmed using subcellular fractionation and immunoblotting (Fig. 9g, h). Again, the YAP nuclear/cytoplasmic ratio was reduced by approximately 50 percent in DDR2-deficient cells regardless of BMP2 status. Levels of phosphorylated YAP (P-YAP S127) measured on immunoblots also increased in Ddr2-deficient cells in both the presence or absence of BMP2 (Fig. 9i, j), which suggests that LATS1/2 activity was elevated in the absence of DDR2. This may in part explain the reduction in nuclear YAP. In summary, DDR2 stimulates the nuclear accumulation of YAP/TAZ, and this may explain its function in BMP2-dependent gene expression and osteoblast differentiation.

## Discussion

Our understanding of accessory factors necessary for BMP-induced osteogenesis is quite limited. This has undoubtedly had a major impact on the success of BMP therapeutics for treating bone disorders. A long-standing paradox in the BMP field has been the disparity between BMP concentrations detected in vivo during development and necessary to directly stimulate cellular responses (normally in the ng/mL range) versus concentrations required to stimulate clinical regeneration (in the mg/mL range)^3,5,42^. The ability of the collagenous ECM to enhance the sensitivity of cells to BMPs may, at least in part, explain this disparity between local cellular responses to BMP and the insensitivity of tissues to therapeutic BMP administration. Until now, however, the identification of the collagen receptors mediating this response to ECM was unclear. Early studies suggested a role for the collagen-binding integrins in BMP responsiveness of preosteoblasts in cell culture although the involvement of integrins has not been evaluated in an in vivo model of BMP-induced bone formation^9,12,15^. In contrast, the present study clearly establishes an important role for DDR2 in BMP activity in vivo and identifies connective tissue-associated GLI1^+^ progenitor cells as an important cellular target for DDR2 regulation of BMP activity. We also show that *Ddr2* deficiency attenuates the HO induced by an ACVR1 mutant associated with FOP, a finding with potential implications for the management of this disorder. Evidence is also presented that DDR2 is not required for early canonical BMP signaling and may instead function through an indirect mechanism involving the control of YAP/TAZ subcellular localization. Together, these studies provide important insights into the function of DDR2 in BMP-induced osteogenesis and HO and provide a new understanding of the crosstalk between ECM signals and BMP activity. This information may be useful both for the treatment of genetic and trauma-induced HO as well as provide a means of augmenting BMP activity by activation of DDR2.

Based on current evidence, it is likely that DDR2 largely functions in progenitor cell populations where it controls proliferation and differentiation to mature skeletal cells during normal bone development as well as during BMP-induced osteogenesis. During development, lineage tracing of *Ddr2*-expressing cells using TAM-treated *Ddr2*^*mer-icre-mer*^*; R26R*^*tdtomato*^ mice showed tdTomato localization to skeletal regions enriched in progenitors (resting and proliferating chondrocytes of the growth plate and cranial base synchondroses, calvarial sutures) at early times with the label gradually appearing in more mature cell populations (hypertrophic chondrocytes, osteoblasts, and osteocytes) at later times. A majority of DDR2^+^ cells in these studies colocalized with the stem cell marker, GLI1^19,20^. In the present study, colocalization of DDR2 and GLI1 was demonstrated in both control and BMP2 implants. Also, tdTomato^+^ cells in *Ddr2*^*mer-icre-mer*^*; R26R*^*tdtomato*^ mice were initially detected in connective tissue adjacent to BMP implants where they stained with GLI1 antibody. As ectopic bone formation progressed, tdTomato^+^ cells migrated into implants where they were first associated with the chondrocyte marker, SOX9, followed by colocalization with the osteoblast/preosteoblast marker, OSX (Figs. 2, 3). Also consistent with a function in GLI1^+^ progenitors, selective inactivation of *Ddr2* in *Gli1*-expressing cells during development using *Gli1*^*CreERT*^*; Ddr2*^*fl/fl*^ mice resulted in a very similar phenotype to that seen in globally *Ddr2*-deficient (*Ddr2*^*slie/slie*^) animals. Specifically, both the conditional and global knockout animals were dwarf, had defects in the growth plate and synchondrosis cartilage proliferation, and exhibited unfused prefrontal sutures^19,20^. Similarly, in the present study BMP2-induced ectopic bone formation was reduced by approximately 50 percent in both *Ddr2*^*slie/slie*^ and *Gli1*^*CreERT*^*; Ddr2*^*fl/fl*^ mice. Also, as shown in lineage tracing experiments using *Gli1-Cre*^*ERT*^*; R26R*^*tdTomato*^ and *Gli1-Cre*^*ERT*^*; Ddr2*^*fl/fl*^*; R26R*^*tdTomato*^ mice, this inhibition was explained by a specific reduction in proliferation and differentiation of a GLI1-derived cell population that migrated into BMP2 implants where they were major contributors to subsequent bone formation (Figs. 4, 5). In contrast, loss of DDR2 did not affect apoptosis although this parameter was inhibited by BMP treatment. In prior studies, BMPs have been reported to either inhibit or stimulate apoptosis depending on the experimental system^43,44^.

A skeletal progenitor-related DDR2 function may also explain the requirement for this collagen receptor in genetic and trauma-induced forms of HO. In the present study, selective inactivation of *Ddr2* in PDGFRA^+^ fibro/adipogenic progenitor cells inhibited the HO induced by an R206H FOP ACVR1 mutant by approximately 50 percent. Similarly, in an earlier collaborative study inactivation of *Ddr2* in PDGFRA^+^ cells was shown to partially inhibit HO caused by a systemic trauma (burn injury) combined with surgical resection of the Achilles tendon^45^. Both these studies inactivated *Ddr2* in PDGFRA^+^ cells while in BMP2 studies *Ddr2* was inactivated in GLI1^+^ cells. Both GLI1 and PDGFRA are expressed in skeletal progenitor cells of mesenchymal origin^6,27,30^, but the precise relationship between these two cell types is not known. Therefore, we conducted lineage tracing studies to explore the relationship between GLI1^+^ and PDGFRA^+^ cells and their progeny during BMP2-induced HO. Interestingly, although both cell types participated in BMP-induced bone formation, the relative contribution of GLI1^+^ cells was much greater than PDGFRA^+^ cells (compare tdTomato fluorescence in Fig. 5b, c with Fig. S5b). Consequently, when *Ddr2* was inactivated using *Pdgfra*^*CreER*^, HO was reduced by only 20 percent while inactivation with *Gli1*^*CreERT*^ reduced mineralization by 50 percent. As indicated by these results, *Ddr2* functions in GLI1^+^ and PDGFRA^+^ cells and their progeny where it is necessary for maximal HO formation. However, because PDGFRA^+^ cells represent only a small fraction of the cells participating in BMP2-induced HO formation, *Ddr2* inactivation in this population reduces HO less than when it is inactivated in GLI1^+^ cells which include most if not all BMP2 responsive cells. In contrast, in the FOP model, aberrant BMP signaling is only induced in PDGFRA^+^ cells where the *Pdgfra*^*CreERT*^ both activates the ACVR1 mutation and inactivates *Ddr2*. In this case, since there is no added BMP2 ligand to activate signaling in non-PDGFRA^+^ cells, all the effects of *Ddr2* inactivation are restricted to PDGFRA^+^ cells and their progeny, which, in the case of FOP, is the main cell lineage responsible for HO^35^. Based on these results, we conclude that *Ddr2* has similar effects in FOP and BMP-induced HO, but in the FOP model, the HO response is restricted to PDGFRA^+^ cells, which contribute less to BMP-induced HO than to FOP-associated HO.

Notably, in both the BMP and FOP-induced ectopic bone formation models used in this study, *Ddr2* inactivation reduced HO by approximately 50 percent. Also, as previously reported, *Ddr2* inactivation only partially inhibits trauma-induced HO^45^. In no case could this partial inhibition be explained by inefficient inactivation of the *Ddr2* allele since only very low DDR2 immunofluorescence was observed in Cre-expressing mice. Therefore, the residual mineralization observed with conditional *Ddr2* knockout must be independent of DDR2 activity. The factor/s responsible for the remaining HO are currently unknown. As noted in the Introduction, BMP-induced osteoblast differentiation requires the binding of SPCs to collagen. Although the other vertebrate DDR, DDR1, is a potential mediator of this interaction, it is expressed at very low levels in connective tissues where HO is occurring making it unlikely that it plays a major role^45^. Instead, we speculate that collagen-binding β1 integrins account for much of the residual osteogenic response. Several prior studies have implicated β1 integrins in bone development and BMP responsiveness. For example, *Itgb1* inactivation in skeletal progenitor cells and chondrocytes is associated with severe axial and craniofacial skeletal deficits and defective fracture healing^15,46–49^. Also, BMP-induced osteoblast differentiation is reduced in cells from *Itgb1*-deficient mice^15^ while blocking antibodies to α1 and α2 integrins disrupt BMP-dependent osteoblast differentiation^9^. There is also evidence from other systems showing that DDR2 and integrins cooperatively interact to interpret ECM signals. For example, in cancer-associated fibroblasts, DDR2 activates β1 integrin activity via RAP1 stimulation of Talin1 and Kindlin2 to promote breast tumor metastasis^38^. Also, the related DDR, DDR1b, colocalizes with β1 integrin and talin in focal adhesions where it stimulates Rac and cell migration^50^. Further studies will be required to determine the degree to which integrins account for BMP-induced heterotopic bone formation.

**Fig. 10:**
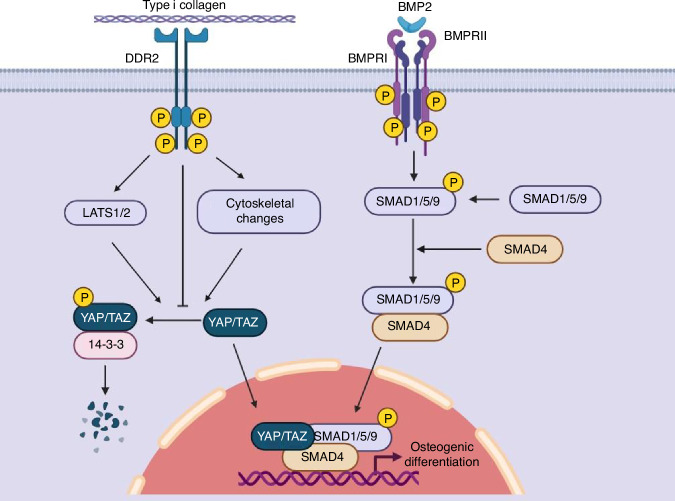
Proposed model for Regulation of BMP Activity by DDR2. After binding collagen, DDR2 alone or together with collagen-binding β1 integrins stabilizes YAP/TAZ nuclear accumulation. This is accomplished in part by inhibiting YAP S127 phosphorylation possibly by inhibiting LATS1/2, thereby inhibiting cytoplasmic degradation, and could also involve increased actin polymerization and cytoskeletal stiffening leading to increased nuclear YAP/TAZ retention. BMP2 stimulates SMAD1/5/9 phosphorylation thereby promoting interaction with SMAD4 and nuclear accumulation of an R-SMAD-SMAD4 complex. In the nucleus, the WW1 domain of YAP binds and stabilizes the R-SMAD-SMAD4 complex to stimulate BMP-dependent transcription of genes necessary for cartilage and bone formation

A major question raised by our studies relates to the mechanism used by DDR2 to regulate BMP activity. Although we show DDR2 is important for BMP2-induced ectopic bone formation in vivo and for in vitro differentiation of BMSCs, we found no evidence that it is required for early BMP signaling related to SMAD phosphorylation and nuclear retention and only saw minor changes in stimulation of noncanonical BMP pathways (MAPK or AKT). Instead, *Ddr2*-deficient mice and BMSCs showed reduced levels of nuclear YAP and TAZ. In BMSCs, these changes were accompanied by a modest increase in YAP phosphorylation. This suggests that DDR2 can inhibit LATS1/2 mediated phosphorylation and proteasomal degradation of YAP/TAZ to promote their nuclear accumulation. While some differences were seen between in vivo and cell culture results (i.e., Ddr2 deficiency reduced total YAP in vivo, but not in cell culture. BMP2 increased nuclear YAP in vivo, but not in cell culture), Ddr2 was shown to be required for nuclear YAP accumulation in both experimental systems. These results are consistent with a previous report from our group where DDR2 was shown to increase nuclear YAP and TAZ levels during trauma-induced HO^45^. How might a DDR2-dependent accumulation of nuclear YAP/TAZ explain the involvement of DDR2 in the BMP response? Our current model for DDR2 actions is summarized in Fig. 10. As shown previously, YAP binds R-SMADs via its WW1 domain, resulting in SMAD stabilization and increased transcriptional activity^51–53^. Thus, one can envision a mechanism where DDR2, alone or together with collagen-binding integrins, responds to the collagenous ECM by inhibiting LATS1/2 to stabilize YAP and TAZ. In addition, DDR2 and integrin signals may stimulate actin polymerization and cytoskeletal stiffness leading to increased nuclear YAP/TAZ retention, which, in the presence of a BMP stimulus, stabilizes nuclear SMADS1/5/9 leading to increased transcription of genes necessary for cell proliferation, chondrocyte and osteoblast differentiation and bone formation.

Although we find the above hypothesis to be an attractive explanation for how DDR2 regulates the BMP response, other DDR2 activities may also be important. For example, DDR2, via its intrinsic receptor tyrosine kinase activity, can stimulate MAP kinase-dependent phosphorylation of the RUNX2 transcription factor and osteoblast-specific gene expression, a response that can be amplified by BMP2^18,54,55^. Further studies will be required to determine if these or related mechanisms adequately explain the observed effects of DDR2 on BMP signaling.

## Materials and Methods

### Mice

All mouse studies were approved by the University of Michigan Committee on the Use and Care of Animals and conformed to all guidelines and regulations for the protection of animal subjects. Mice were group housed in specific pathogen-free AAALAC-certified facilities at 23–25 °C with a 12 h light/dark cycle and allowed free access to water and standard laboratory chow. Globally Ddr2-deficient (*Ddr2*^*slie/slie*^) mice, which contain a spontaneous 150 kb deletion in the *Ddr2* locus to produce an effective null, were initially obtained from the Jackson Laboratory and bred with C57BL/J6 (B6) mice for at least 10 generations^18^. *Ddr2*^*fl/fl*^ mice with *loxP* sites flanking coding exon 8 of the *Ddr2* gene and *Ddr2*^*mer-iCre-mer*^ mice harboring a MerCreMer cassette knocked in-frame into exon 2 of the *Ddr2* locus were previously described^19,56^. For lineage analysis, *Rosa26*^*LSL-tdTomato*^ mice^57^ were crossed with either *Ddr2*^*mer-iCre-mer*^ mice to generate *Ddr2*^*mer-iCre-mer*^; *Rosa26*^*LSL-tdTomato*^ mice or with *Gli1*^*CreERT*^ mice^58^ to generate *Gli1*^*CreERT*^*; Rosa26*^*LSL-tdTomato*^ mice. Conditional inactivation of *Ddr2* in GLI1-positive progenitor cells was achieved by crossing *Ddr2*^*fl/fl*^ mice with *Gli1*^*CreERT*^ mice to generate *Gli1*^*CreERT*^; *Ddr2*^*fl/fl*^ mice or by crossing *Gli1*^*CreERT*^*; Rosa26*^*LSL-tdTomato*^ mice with *Ddr2*^*fl/fl*^ mice to generate *Gli1*^*CreERT*^; *Ddr2*^*fl/fl*^; *Rosa26*^*LSL-tdTomato*^ mice. Conditional inactivation of *Ddr2* in PDGFRA-positive progenitor cells was achieved by crossing *Pdgfra*^*CreER*^ mice^59^ with *Ddr2*^*fl/fl*^; *Rosa26*^*LSL-tdTomato*^ mice to generate *Pdgfra*^*CreER*^; *Ddr2*^*fl/fl*^; *Rosa26*^*LSL-tdTomato*^ mice. For Cre activation, mice received i.p. tamoxifen (TAM) injections at 50 mg/kg for 3 consecutive days prior to surgery. BMSC implant studies used immunodeficient mice (Charles River Labs, CB17/lcr-*Prkdc*^*scid*^/lcrlcoCrl)

To evaluate the effects of *Ddr2* status in a mouse model of FOP^33^, *Pdgfra*^*CreER*^*; Acvr1*^*[R206H]FlEx/+*^*; Ddr2*^*fl/fl*^ and *Pdgfra*^*CreER*^*; Acvr1*^*[R206H]FlEx/+*^*; Ddr2*^*fl/+*^ mice were generated by crossing *Pdgfra*^*CreER*^*; Acvr1*^*[R206H]FlEx/+*^ and *Ddr2*^*fl/fl*^ mice. The induction protocol was previously described^34^. Briefly, eight to 10-week-old animals were treated i.p. with tamoxifen (75 mg/kg) twice a week for 3.5 weeks (total of 7 tamoxifen injections). After an additional 3.5 weeks, the lower hindlimb was injected with cardiotoxin (0.3 μg/site) to induce muscle damage and limbs were harvest after 14 days to monitor HO formation by μCT and histology.

### Genotyping

Genomic DNA was prepared from tail snips or ear punches using DNA REDExtract-N-Amp™ Tissue PCR Kit (Sigma-Aldrich, St. Louis, MO). Polymerase chain reaction (PCR) analysis was used for genotyping of Ddr2^fl/f^, *Ddr2*^*Mer-icre-Mer*^, *Gli1*^*CreERT*^, and *R26R*^*TdTomato*^ mice. Primer sequences were previously described^20^. The genotyping of Ddr2^slie/slie^ mice was performed by qRT-PCR using conditions defined by Jackson Labs. Primers and conditions for genotyping *Pdgfra*^*CreER*^*; Acvr1*^*[R206H]FlEx/+*^ were previously described^34^.

### Subcutaneous ectopic bone formation model

For BMP2 implants, 1 μg of recombinant BMP2 (a generous gift from Dr. Christopher Wilson, Bioventus) was adsorbed to 175 mm^3^ gelatin sponges (Gelfoam; Upjohn, Kalamazoo, MI, USA) and lyophilized overnight before use. For cell implants, BMSCs were isolated and treated as described below and 2 × 10^6^ cells were adsorbed to gelatin sponges. Ten to 12-week-old mice of the indicated genotypes (or immunodeficient mice for cell implants) were injected with carprofen (5 mg/kg) before surgery. Mice were then anesthetized using inhaled isoflurane. Skin incisions of about 1 cm in length were made in the center of the back. Small pouches were then bluntly dissected in the subcutaneous tissue, and gelatin sponges containing BMP2 or BMSCs were implanted. Two implants were placed per mouse (1 per side). Wounds were closed with surgical staples to facilitate the removal of the implant, which was harvested after up to 4 weeks. As shown in control dosing experiments, 1 μg BMP2/implant was sufficient to form a complete ossicle containing cortical and trabecular bone (Fig. S8a, b).

### BMSC cell culture and in vitro treatment protocols

BMSC were isolated from femurs and tibias of 10–12-week-old *Ddr2*^*fl/fl*^ mice as previously described^18^. Confluent cultures were trypsinized and plated at a density of 50 000 cells/cm^2^ and transduced with control (Ad-lacZ) or Cre-expressing adenovirus (Ad-Cre) using a titer of 600 pfu/cell in serum-free medium. After overnight incubation, 20% FBS αMEM medium was added to replace serum-free medium, and cells were grown for an additional 48 h. Cells were used for in vivo implants or cell culture analysis of osteoblast differentiation. In the latter case, BMSCs were grown in α-MEM/10%FBS containing 50 μg/mL ascorbic acid and 10 mmol/L β-glycerophosphate with or without 100 ng/mL BMP2. Mineral deposition was assessed by Alizarin Red staining and quantified as previously described^60^. The BMP2 dose selected was shown to optimally stimulate osteoblast differentiation and mineralization (Fig. S8c, d).

For acute BMP2 treatment studies, undifferentiated BMSCs were transduced with Ad-lacZ or Ad-Cre and then grown in 0.1% FBS αMEM medium overnight. Cells were treated with BMP2 (100 ng/mL) to stimulate early BMP2 signaling and harvested after increasing times up to two hours.

### RNA analysis

Total RNA was collected from BMSC cultures using trizol and RNeasy kit (QIAGEN Hilden, Germany). A Taqman Reverse Transcription Kit (Applied BioSystems, Inc, Foster City, CA, USA) was used to reverse transcribe mRNA into cDNA. The power SYBR Green PCR Master Mix Kit (Applied BioSystems, Inc) was used for quantitative real-time PCR (qRT-PCR). The following Taqman probes (Applied Biosystems) were used: glyceraldehyde-3-phosphate dehydrogenase (*Gapdh,* Mm99999915), *Osteocalcin* (βglap, Mm03413826), *Bone sialoprotein* (*Ibsp*, Mm01208378) and Alkaline phosphatase (*Alpl*, Mm00475834). *Gapdh* was used to normalize target gene expression levels.

### Micro-computed tomography analysis

Following euthanasia, ossicles were collected and fixed with 4% PBS-buffered paraformaldehyde at 4 °C overnight followed by by micro-computed tomography analysis using a Scanco Model 100 (Scanco Medical, Bassersdorf, Switzerland). Scanning settings were as follows: voltage 80 kVp, current 80 μA, exposure time 1 600 ms, voxel size in the reconstructed image 18 μm, isotropic. The data were processed and analyzed using MicroView (v2.1.2 Advanced Bone Application; GE Healthcare Preclinical Imaging). A threshold of 180 for trabecular bone analysis was used throughout the whole study and used to determine bone volume (BV) and bone volume/total volume (BV/TV). The mouse genotype was not specified during μCT analysis to avoid examiner bias.

### Proliferation and apoptosis assays

To measure cell proliferation, mice were injected intraperitoneally with 5-ethynyl-2′-deoxyuridine (EdU, Invitrogen, # C10337) 24 h before harvest. EdU-labeled cells were detected using Click-iT® EdU Alexa Fluor® 488 Imaging Kit (Invitrogen, # C10337) according to the manufacturer’s instructions. Sections *were* mounted using ProLong™ Gold Antifade Mountant containing 4′,6-diamidino-2-phenylindole (DAPI, Life technologies) for cell nuclei staining. In some studies, proliferation was assessed by immunofluorescence staining for P-Histone H3 (see below). For apoptosis, a CF^®^488 TUNEL Assay apoptosis Detection kit was used (Biotium, 30063). Sections were mounted with DAPI for cell nuclei staining.

### Histology and immunofluorescence analysis

Implants were harvested and fixed in 4% PFA overnight followed by decalcification in 10% EDTA (pH 7.4) for 2 weeks. Samples were processed for either cryosections (14 μm) or paraffin sections (7 μm). For histological analysis, samples were stained with hematoxylin and eosin (paraffin sections) or Safranin O/Fast Green (frozen sections) according to standard procedures.

For immunofluorescence staining, cryosections were placed in a cold fixative (4% PFA) for 10 min. Sections were then permeablized with 0.1% Triton X-100 and subjected to heat-induced antigen retrieval using Diva Decloaker (1:10, DV2004MX, Dako) following the manufacturer's instructions. Sections were blocked with donkey-serum blocking solution (10% donkey serum, 0.1% Triton X-100) for 2 h at room temperature (RT) and then incubated at 4 °C overnight with the following primary antibodies: DDR2 (LS Bio B15752, 1:200), SOX9 (ab185230, 1:100), SP7 (ab22552, 1:100), Gli1 (AF3455, 1:40), p-Histone H3 S10 (ZRB1226, 1:100). IBSP polyclonal antibody was produced in the project laboratory and used at a 1:200 dillution^20^. Slides were washed in 1× PBS three times and incubated with fluorescence-conjugated secondary antibodies for 2 h at RT (Invitrogen donkey anti-Rabbit AF488, Invitrogen donkey anti-goat AF488, Invitrogen donkey anti-goat AF568, all secondary antibodies at 1:1 000 dilution). Slides were mounted using ProLong™ Gold Antifade Mountant with DAPI (Life Technologies) for cell nuclei staining. For fluorescent immunocytochemistry, BMSCs were fixed in 4% PFA and permeablized with 0.1% Triton X-100, blocked with donkey-serum blocking solution, and incubated at 4 °C overnight with the following primary antibodies: P-SMAD1/5 (Cell Signaling Technology, 1:100), TAZ (BD Biosciences, 560235, 1:500), YAP (Cell Signaling Technology, 14074 s 1:200). Cells were incubated with species-matched secondary antibody (Invitrogen donkey anti-Rabbit AF488) for 1 h at RT. The slides were mounted using ProLong™ Gold Antifade Mountant with DAPI (Life Technologies, P36930) for cell nuclei staining. For most studies, images were acquired with an Eclipse E800 microscope (Nikon). Quantification of fluorescence was accomplished using FIJI software (https://imagej.nih.gov/ij/download.html) with Colocalization Image Creator and Colocalization Object Counter plugins. For analysis of YAP distribution in vivo, images were acquired and analyzed with a Leica DMi8 microscope with Thunder imaging system (Leica Application Suite X software).

### Immunoblot analysis

Western blotting was performed using standard procedures. Cultured cells were lysed in RIPA buffer (Thermo Fisher Scientific, 89900) and freshly supplemented with protease and phosphatase inhibitor cocktails (Thermo Fisher Scientific 78442). Protein concentration was determined by a BCA protein assay kit (Pierce Biotech, Rockford, IL), and equal amounts of protein were resolved by 10% sodium dodecylsulfate–polyacrylamide gel electrophoresis (SDS–PAGE) and transferred to nitrocellulose membranes (Amersham Biosciences, Buckinghamshire, UK). After blocking in Tris-buffered saline containing 5% powdered milk and 0.1% Tween-20, membranes were incubated with primary antibodies, followed by incubation with horseradish peroxidase (HRP)-labeled secondary antibodies. The following primary antibodies and dillutions were used: DDR2 (EMD Millipore MABT322, 1: 1 000); GAPDH (Cell Signaling Technology 2118 L, 1:1 000), phospho-SMADs1/5 (Cell Signaling Technology 9516 s, 1:1 000), total SMAD1 (Cell Signaling Technology 6944ss, 1:1 000), phospho-ERK1/2 (Cell Signaling Technology 9101 s, 1:1 000), total ERK1/2 (Cell Signaling Technology 9102 s, 1:1 000), phospho-AKT (Cell Signaling Technology 4060 s, 1:1 000), total AKT (Cell Signaling Technology 9272, 1:1 000), phospho-p38 (Cell Signaling Technology 9211 s, 1:1 000) and total p38 (Cell Signaling Technology 9212 s, 1:1 000), YAP (Cell Signaling Technology 14074 s, 1:1 000), Lamin A/C (Cell Signaling Technology 4777 s, 1:1 000) and P-YAP-S127 (Abcam ab76252). For subcellular fractionation studies, NE-PERTM Nuclear and Cytoplasmic Extraction Reagents were used (Thermo Fisher, #78835). In preliminary experiments, molecular weights of all proteins on immunoblots were verified with protein standards (not shown).

### Statistical analysis

All data were analyzed using GraphPad Prism software (version 9.0, La Jolla, CA, USA).

According to the number of independent variables and groups, a two-way ANOVA or two-tailed unpaired t-test was used to evaluate statistical significance. A sample size of at least 6 mice for each experimental group was used for in vivo experiments measuring ectopic bone formation and at least three mice were used for histological analysis. For cell culture studies, triplicate-independent samples were used for all analyses. Results are presented as mean ± SD as indicated. *P*-values are designated as follows: *P < 0.05; **P < 0.01, ***P < 0.001. ns, not significant at *P* < 0.05.

## Supplementary information

Wu et al. Supplementary Materials

## Data Availability

The data supporting the findings of this study are available from the corresponding author upon reasonable request.

## References

[1] Grafe, I. et al. TGF-β family signaling in mesenchymal differentiation. *Cold Spring Harb. Perspect. Biol.* **10**, a022202 (2018). 10.1101/cshperspect.a022202 PMC5932590 28507020

[2] Salazar, V. S., Gamer, L. W. & Rosen, V. BMP signalling in skeletal development, disease and repair. *Nat. Rev. Endocrinol.* **12**, 203–221 (2016). 26893264 10.1038/nrendo.2016.12

[3] Cahill, K. S., McCormick, P. C. & Levi, A. D. A comprehensive assessment of the risk of bone morphogenetic protein use in spinal fusion surgery and postoperative cancer diagnosis. *J. Neurosurg. Spine* **23**, 86–93 (2015). 25860517 10.3171/2014.10.SPINE14338

[4] Ali, I. H. & Brazil, D. P. Bone morphogenetic proteins and their antagonists: current and emerging clinical uses. *Br. J. Pharmacol.* **171**, 3620–3632 (2014). 24758361 10.1111/bph.12724 PMC4128061

[5] Giannoudis, P. V. & Einhorn, T. A. Bone morphogenetic proteins in musculoskeletal medicine. *Injury* **40**, S1–S3 (2009). 20082783 10.1016/S0020-1383(09)00642-1

[6] Meyers, C. et al. Heterotopic ossification: a comprehensive review. *JBMR* **3**, e10172 (2019). 10.1002/jbm4.10172 PMC6478587 31044187

[7] Hwang, C. D. et al. Contemporary perspectives on heterotopic ossification. *JCI Insight* **7**, e158996 (2022). 10.1172/jci.insight.158996 PMC9431693 35866484

[8] Xiao, G. et al. Bone morphogenetic proteins, extracellular matrix, and mitogen-activated protein kinase signaling pathways are required for osteoblast-specific gene expression and differentiation in MC3T3-E1 cells. *J. Bone Miner. Res.* **17**, 101–110 (2002). 11771655 10.1359/jbmr.2002.17.1.101

[9] Jikko, A., Harris, S. E., Chen, D., Mendrick, D. L. & Damsky, C. H. Collagen integrin receptors regulate early osteoblast differentiation induced by BMP-2 [In Process Citation]. *J. Bone Miner. Res.* **14**, 1075–1083 (1999). 10404007 10.1359/jbmr.1999.14.7.1075

[10] da Silva Madaleno, C., Jatzlau, J. & Knaus, P. BMP signalling in a mechanical context - Implications for bone biology. *Bone* **137**, 115416 (2020). 32422297 10.1016/j.bone.2020.115416

[11] Ashe, H. L. Modulation of BMP signalling by integrins. *Biochem. Soc. Trans.* **44**, 1465–1473 (2016). 27911728 10.1042/BST20160111

[12] Lai, C. F. & Cheng, S. L. Alphavbeta integrins play an essential role in BMP-2 induction of osteoblast differentiation. *J. Bone Miner. Res.* **20**, 330–340 (2005). 15647827 10.1359/JBMR.041013

[13] Suzawa, M. et al. Stimulation of Smad1 transcriptional activity by Ras-extracellular signal-regulated kinase pathway: a possible mechanism for collagen-dependent osteoblastic differentiation. *J. Bone Miner. Res.* **17**, 240–248 (2002). 11811554 10.1359/jbmr.2002.17.2.240

[14] Xiao, G., Wang, D., Benson, M. D., Karsenty, G. & Franceschi, R. T. Role of the alpha2-integrin in osteoblast-specific gene expression and activation of the Osf2 transcription factor. *J. Biol. Chem.* **273**, 32988–32994 (1998). 9830051 10.1074/jbc.273.49.32988

[15] Brunner, M. et al. beta1 integrins mediate the BMP2 dependent transcriptional control of osteoblast differentiation and osteogenesis. *PLoS One* **13**, e0196021 (2018). 29677202 10.1371/journal.pone.0196021 PMC5909894

[16] Shekaran, A. et al. Bone regeneration using an alpha 2 beta 1 integrin-specific hydrogel as a BMP-2 delivery vehicle. *Biomaterials* **35**, 5453–5461 (2014). 24726536 10.1016/j.biomaterials.2014.03.055 PMC4033404

[17] Franceschi, R. T., Hallett, S. A. & Ge, C. Discoidin domain receptors; an ancient family of collagen receptors has major roles in bone development, regeneration and metabolism. *Front. Dent. Med*. **4**, 1181817 (2023). 10.3389/fdmed.2023.1181817 PMC10785288 38222874

[18] Ge, C. et al. Discoidin receptor 2 controls bone formation and marrow adipogenesis. *J. Bone Miner. Res.* **31**, 2193–2203 (2016). 27341689 10.1002/jbmr.2893 PMC5135576

[19] Mohamed, F. F. et al. The collagen receptor, discoidin domain receptor 2, functions in Gli1-positive skeletal progenitors and chondrocytes to control bone development. *Bone Res.* **10**, 11 (2022). 35140200 10.1038/s41413-021-00182-w PMC8828874

[20] Mohamed, F. F. et al. Control of craniofacial development by the collagen receptor, discoidin domain receptor 2. *Elife* **12**, e77257 (2023). 10.7554/eLife.77257 PMC9977278 36656123

[21] Binrayes, A., Ge, C., Mohamed, F. F. & Franceschi, R. T. Role of discoidin domain receptor 2 in craniofacial bone regeneration. *J. Dent. Res.* **100**, 1359–1366 (2021). 33899571 10.1177/00220345211007447 PMC8532241

[22] Binrayes, A., Ge, C., Mohamed, F., Kozloff, K. M. & Franceschi, R. T. Role of discoidin domain receptor 2 in bone regeneration. *J. Bone Miner. Res.* **33**, S0448 (2018).

[23] Bok, S. et al. A multi-stem cell basis for craniosynostosis and calvarial mineralization. *Nature* **621**, 804–812 (2023). 10.1038/s41586-023-06526-2 PMC10799660 37730988

[24] Bargal, R. et al. Mutations in DDR2 gene cause SMED with short limbs and abnormal calcifications. *Am. J. Hum. Genet.* **84**, 80–84 (2009). 19110212 10.1016/j.ajhg.2008.12.004 PMC2668047

[25] Ge, C., Li, Y., Wu, F., Ma, P. & Franceschi, R. T. Synthetic peptides activating discoidin domain receptor 2 and collagen-binding integrins cooperate to stimulate osteoblast differentiation of skeletal progenitor cells. *Acta Biomater*. **166**, 109–118 (2023). 10.1016/j.actbio.2023.05.039 PMC10617013 37245640

[26] Wang, Q., Huang, C., Xue, M. & Zhang, X. Expression of endogenous BMP-2 in periosteal progenitor cells is essential for bone healing. *Bone* **48**, 524–532 (2011). 21056707 10.1016/j.bone.2010.10.178 PMC3039040

[27] Kan, C. et al. Gli1-labeled adult mesenchymal stem/progenitor cells and hedgehog signaling contribute to endochondral heterotopic ossification. *Bone* **109**, 71–79 (2018). 28645539 10.1016/j.bone.2017.06.014 PMC5801258

[28] Wosczyna, M. N., Biswas, A. A., Cogswell, C. A. & Goldhamer, D. J. Multipotent progenitors resident in the skeletal muscle interstitium exhibit robust BMP-dependent osteogenic activity and mediate heterotopic ossification. *J. Bone Miner. Res.* **27**, 1004–1017 (2012). 22307978 10.1002/jbmr.1562 PMC3361573

[29] Agarwal, S. et al. Inhibition of Hif1alpha prevents both trauma-induced and genetic heterotopic ossification. *Proc. Natl. Acad. Sci. USA* **113**, E338–E347(2016). 26721400 10.1073/pnas.1515397113 PMC4725488

[30] Kan, C. et al. BMP-dependent, injury-induced stem cell niche as a mechanism of heterotopic ossification. *Stem Cell Res. Ther.* **10**, 14 (2019). 30635039 10.1186/s13287-018-1107-7 PMC6329163

[31] Lefebvre, V. & de Crombrugghe, B. Toward understanding SOX9 function in chondrocyte differentiation. *Matrix Biol.* **16**, 529–540 (1998). 9569122 10.1016/s0945-053x(98)90065-8

[32] Elmaci, I., Altinoz, M. A., Sari, R. & Bolukbasi, F. H. Phosphorylated histone H3 (PHH3) as a novel cell proliferation marker and prognosticator for meningeal tumors: a short review. *Appl. Immunohistochem. Mol. Morphol.* **26**, 627–631 (2018). 28777144 10.1097/PAI.0000000000000499

[33] Hatsell, S. J. et al. ACVR1R206H receptor mutation causes fibrodysplasia ossificans progressiva by imparting responsiveness to activin A. *Sci. Transl. Med.* **7**, 303ra137 (2015). 26333933 10.1126/scitranslmed.aac4358 PMC6164166

[34] Pan, H., Fleming, N., Hong, C. C., Mishina, Y. & Perrien, D. S. Methods for the reliable induction of heterotopic ossification in the conditional Alk2(Q207D) mouse. *J. Musculoskelet. Neuronal Interact.* **20**, 149–159 (2020). 32131380 PMC7104591

[35] Lees-Shepard, J. B. et al. Activin-dependent signaling in fibor/adipogenic progenitors causes fibrodysplasia ossificans progressiva. *Nat. Commun.* **9**, 471 (2018). 29396429 10.1038/s41467-018-02872-2 PMC5797136

[36] Huang, Y. C., Kaigler, D., Rice, K. G., Krebsbach, P. H. & Mooney, D. J. Combined angiogenic and osteogenic factor delivery enhances bone marrow stromal cell-driven bone regeneration. *J. Bone Miner. Res.* **20**, 848–857 (2005). 15824858 10.1359/JBMR.041226

[37] Mueller, T. D. Mechanisms of BMP-receptor interaction and activation. *Vitam. Horm.* **99**, 1–61 (2015). 26279372 10.1016/bs.vh.2015.06.003

[38] Bayer, S. V. et al. DDR2 controls breast tumor stiffness and metastasis by regulating integrin mediated mechanotransduction in CAFs. *Elife* **8**, e45508 (2019). 31144616 10.7554/eLife.45508 PMC6555593

[39] Hao, J. et al. Role of extracellular matrix and YAP/TAZ in cell fate determination. *Cell Signal.* **26**, 186–191 (2014). 24216612 10.1016/j.cellsig.2013.11.006

[40] Wei, Q. et al. BMP-2 signaling and mechanotransduction synergize to drive osteogenic differentiation via YAP/TAZ. *Adv. Sci.* **7**, 1902931 (2020). 10.1002/advs.201902931 PMC7404154 32775147

[41] Tan, S., Fang, J. Y., Yang, Z., Nimni, M. E. & Han, B. The synergetic effect of hydrogel stiffness and growth factor on osteogenic differentiation. *Biomaterials* **35**, 5294–5306 (2014). 24703716 10.1016/j.biomaterials.2014.02.040

[42] Greenfeld, H., Lin, J. & Mullins, M. C. The BMP signaling gradient is interpreted through concentration thresholds in dorsal-ventral axial patterning. *PLoS Biol.* **19**, e3001059 (2021). 33481775 10.1371/journal.pbio.3001059 PMC7857602

[43] Jiao, G. et al. BMPR2 inhibition induced apoptosis and autophagy via destabilization of XIAP in human chondrosarcoma cells. *Cell Death Dis.* **5**, e1571 (2014). 25501832 10.1038/cddis.2014.540 PMC4649848

[44] Guha, U., Gomes, W. A., Kobayashi, T., Pestell, R. G. & Kessler, J. A. In vivo evidence that BMP signaling is necessary for apoptosis in the mouse limb. *Dev. Biol.* **249**, 108–120 (2002). 12217322 10.1006/dbio.2002.0752

[45] Pagani, C. A. et al. Discoidin domain receptor 2 regulates aberrant mesenchymal lineage cell fate and matrix organization. *Sci. Adv.* **8**, eabq6152 (2022). 36542719 10.1126/sciadv.abq6152 PMC9770942

[46] Shekaran, A. et al. The effect of conditional inactivation of beta 1 integrins using twist 2 Cre, Osterix Cre and osteocalcin Cre lines on skeletal phenotype. *Bone* **68**, 131–141 (2014). 25183373 10.1016/j.bone.2014.08.008 PMC4189988

[47] Aszodi, A., Hunziker, E. B., Brakebusch, C. & Fassler, R. Beta1 integrins regulate chondrocyte rotation, G1 progression, and cytokinesis. *Genes Dev.* **17**, 2465–2479 (2003). 14522949 10.1101/gad.277003 PMC218082

[48] Greer, S. E., Haller, S. J., Lee, D. & Dudley, A. T. N-cadherin and beta1 Integrin coordinately regulate growth plate cartilage architecture. *Mol. Biol. Cell* **35**, ar49 (2024). 10.1091/mbc.E23-03-0101 PMC11064670 38294852

[49] Ekholm, E. et al. Diminished callus size and cartilage synthesis in alpha 1 beta 1 integrin-deficient mice during bone fracture healing. *Am. J. Pathol.* **160**, 1779–1785 (2002). 12000729 10.1016/s0002-9440(10)61124-8 PMC1850876

[50] Borza, C. M. et al. The collagen receptor discoidin domain receptor 1b enhances integrin beta1-mediated cell migration by interacting with talin and promoting Rac1 activation. *Front. Cell Dev. Biol.* **10**, 836797 (2022). 35309920 10.3389/fcell.2022.836797 PMC8928223

[51] Alarcón, C. et al. Nuclear CDKs drive Smad transcriptional activation and turnover in BMP and TGF-beta pathways. *Cell* **139**, 757–769 (2009). 19914168 10.1016/j.cell.2009.09.035 PMC2818353

[52] Huang, Z. et al. YAP stabilizes SMAD1 and promotes BMP2-induced neocortical astrocytic differentiation. *Development* **143**, 2398–2409 (2016). 27381227 10.1242/dev.130658 PMC4958318

[53] Huang, Z. et al. Neogenin promotes BMP2 activation of YAP and Smad1 and enhances astrocytic differentiation in developing mouse neocortex. *J. Neurosci.* **36**, 5833–5849 (2016). 27225772 10.1523/JNEUROSCI.4487-15.2016 PMC4879200

[54] Zhang, Y. et al. An essential role of discoidin domain receptor 2 (DDR2) in osteoblast differentiation and chondrocyte maturation via modulation of Runx2 activation. *J. Bone Miner. Res.* **26**, 604–617 (2011). 20734453 10.1002/jbmr.225

[55] Ge, C. et al. Interactions between extracellular signal-regulated kinase 1/2 and p38 MAP kinase pathways in the control of RUNX2 phosphorylation and transcriptional activity. *J. Bone Miner. Res.* **27**, 538–551 (2012). 22072425 10.1002/jbmr.561 PMC4285380

[56] Mohamed, F. F., Ge, C., Binrayes, A. & Franceschi, R. T. The role of discoidin domain receptor 2 in tooth development. *J. Dent. Res.* **99**, 214–222 (2020). 31869264 10.1177/0022034519892563 PMC7315682

[57] Madisen, L. et al. A robust and high-throughput Cre reporting and characterization system for the whole mouse brain. *Nat. Neurosci.* **13**, 133–140 (2010). 20023653 10.1038/nn.2467 PMC2840225

[58] Ahn, S. & Joyner, A. L. Dynamic changes in the response of cells to positive hedgehog signaling during mouse limb patterning. *Cell* **118**, 505–516 (2004). 15315762 10.1016/j.cell.2004.07.023

[59] Roesch, K. et al. The transcriptome of retinal Muller glial cells. *J. Comp. Neurol.* **509**, 225–238 (2008). 18465787 10.1002/cne.21730 PMC2665263

[60] Zhao, M., Zhao, Z., Koh, J. T., Jin, T. & Franceschi, R. T. Combinatorial gene therapy for bone regeneration: cooperative interactions between adenovirus vectors expressing bone morphogenetic proteins 2, 4, and 7. *J. Cell. Biochem.* **95**, 1–16 (2005). 15759283 10.1002/jcb.20411

