# Supplementary material for: Discoidin domain receptor 2 is an important modulator of BMP signaling during heterotopic bone formation
Source: Bone Res. 2025 Jan 2;13:7. doi: 10.1038/s41413-024-00391-z (PMC11696679; doi:10.1038/s41413-024-00391-z)
Supplement: Supplementary file 1 — Wu et al. Supplementary Materials [file 41413_2024_391_MOESM1_ESM.pdf]

Suppl. Fig1

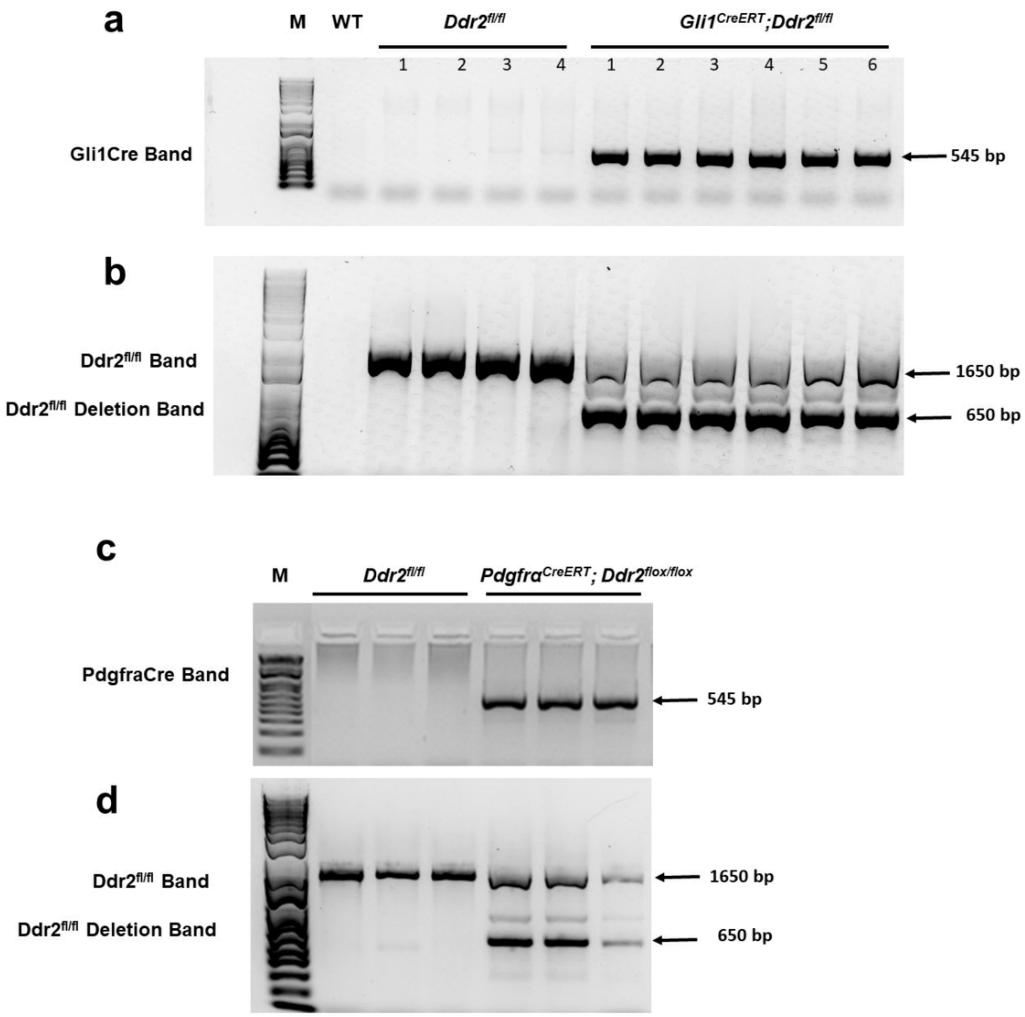

Supplementary Figure 1. Validation of Cre-mediated recombination of *Ddr2*$^{fl/fl}$ allele using *Gli1*^{CreERT} or *Pdgfra*^{CreER}. (a,b) *Ddr2*$^{fl/fl}$ and *Gli1*^{CreERT}*;Ddr2*$^{fl/fl}$ mice were injected with TAM and genomic DNA was isolated from ear punches for PCR analysis. **(c,d)** *Ddr2*$^{fl/fl}$ or *Pdgfra*^{CreER}*;Ddr2*$^{fl/fl}$ mice were injected with TAM and analyzed as described above. Positions of amplified bands for Cre (545 bp), *Ddr2*$^{fl/fl}$ (1650 bp) and the recombined knockout allele (650 bp) are indicated.

Suppl. Fig2

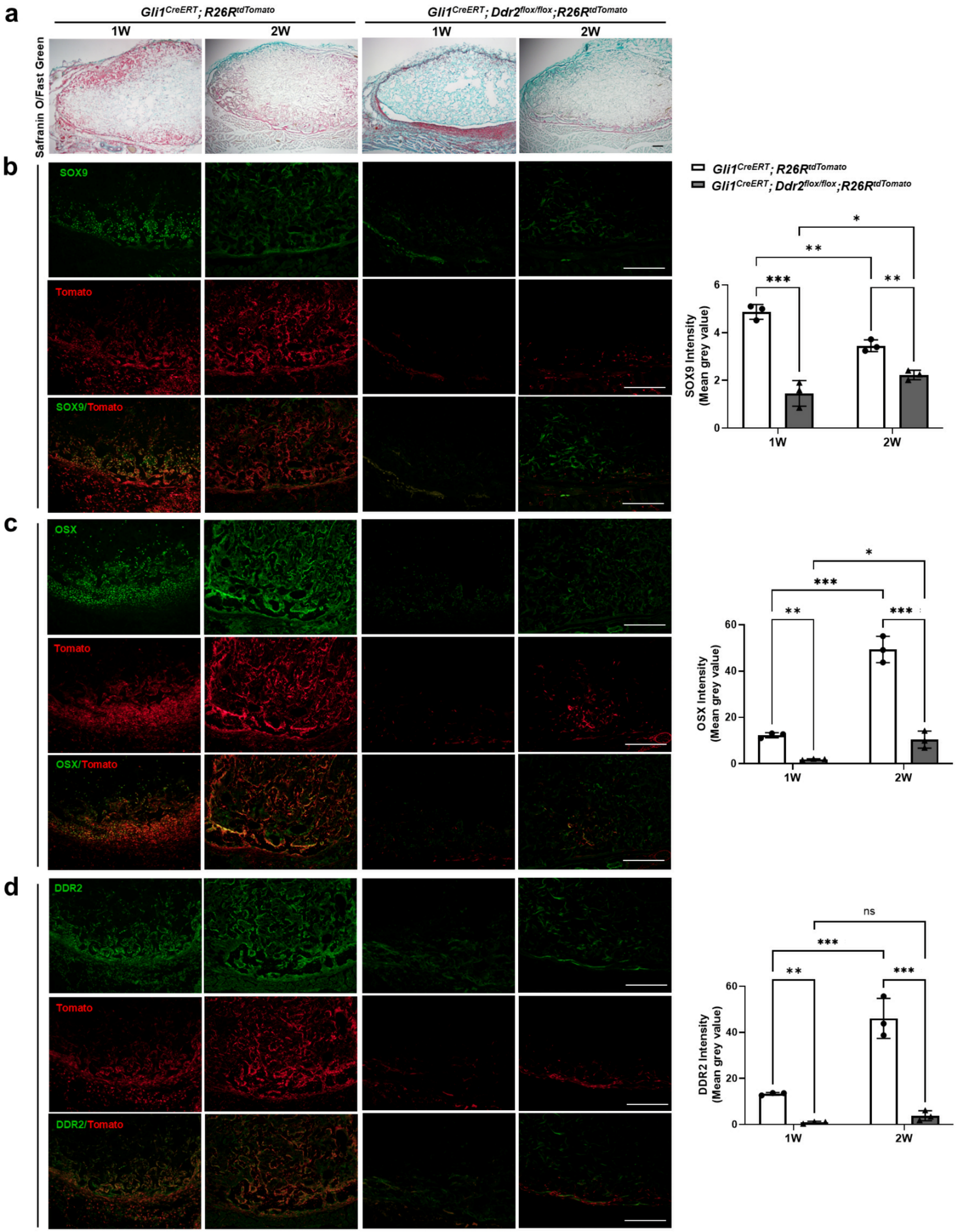

a
Gli1CreERT; R26RtdTomato
Gli1CreERT; Ddr2flox/flox;R26RtdTomato
1W
2W
1W
2W
Safranin O/Fast Green
b
SOX9
Tomato
SOX9/Tomato
Gli1CreERT; R26RtdTomato
Gli1CreERT; Ddr2flox/flox;R26RtdTomato
SOX9 Intensity (Mean grey value)
0
2
4
6
1W
2W
***
**
**
*
c
OSX
Tomato
OSX/Tomato
OSX Intensity (Mean grey value)
0
20
40
60
1W
2W
**
***
***
*
d
DDR2
Tomato
DDR2/Tomato
DDR2 Intensity (Mean grey value)
0
20
40
60
1W
2W
**
***
***
ns

Supplementary Figure 2. Ddr2 inactivation disrupts both chondrogenic and osteogenic phases of endochondral ossification. *Gli1*^{CreERT}*; R26R*^{tdTomato} and *Gli1*^{CreERT}*;Ddr2*$^{fl/fl}$*; R26R*^{tdTomato} mice were treated with tamoxifen, implanted with control or BMP2 implants and examined after 2 days, 1 week or 2 weeks (as in Fig. 5) before measurement of chondrogenesis (Safranin O/Fast Green, **a**; SOX9 IF, **b**) or osteogenesis (OSX IF, **c**). DDR2 IF is also indicated (**d**) as well as tdTomato-positive cell distribution (red). Right panels in **b-d** show quantification of immunofluorescence with SOX9, OSX or DDR2 antibodies respectively. Statistics: two-way ANOVA . *P<0.05, **P<0.01, ***P<0.001, ns not significant. scale bar: 200μm

Suppl. Fig3

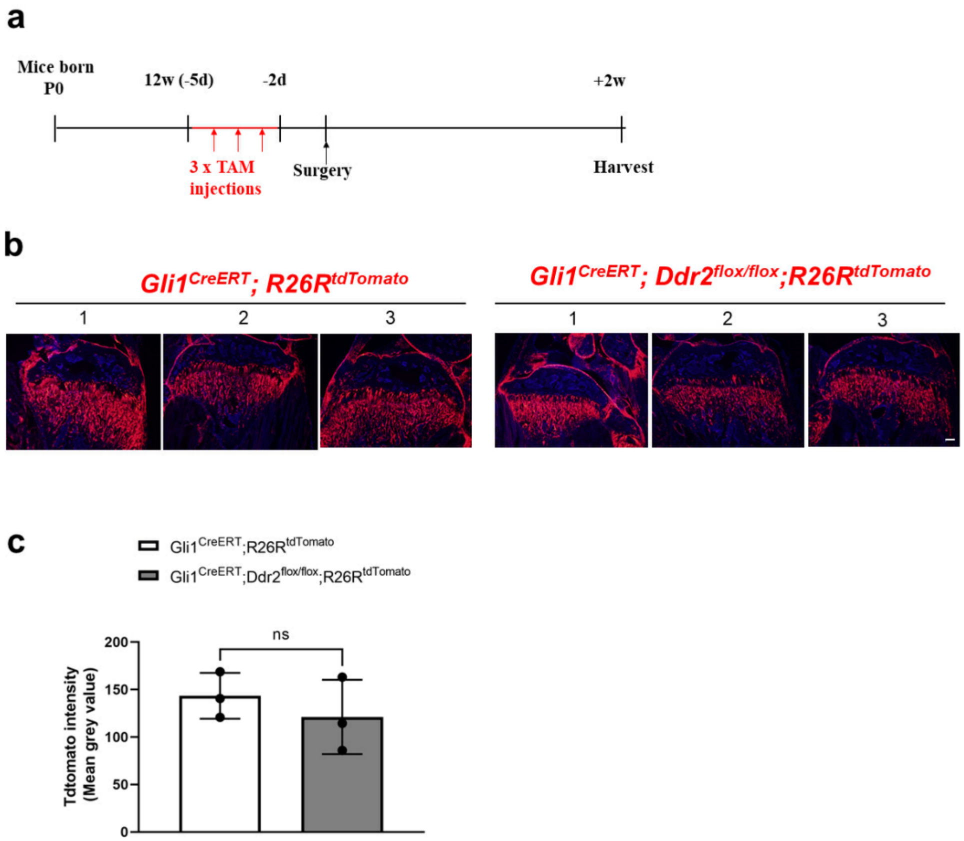

Supplementary Figure 3. Demonstration that *Ddr2* knockout does not affect overall tdTomato expression in *Gli1^{CreERT}; Ddr2$^{fl/fl}$;R26R^{tdTomato}* mouse hosts. Tibias were isolated from the *Gli1^{CreERT}; R26R^{tdTomato}* and *Gli1^{CreERT}; Ddr2$^{fl/fl}$;R26R^{tdTomato}* mice used in the experiment described in Figure 5. Frozen sections were prepared and tdTomato fluorescence was measured. **(a)** Experimental protocol, **(b)** fluorescent images, **(c)** Quantification of tdTomato fluorescence**.** TdTomato fluorescence was not significantly different between the two groups (Two-tailed T test). Scale bar: 200 μm

Suppl. Fig4

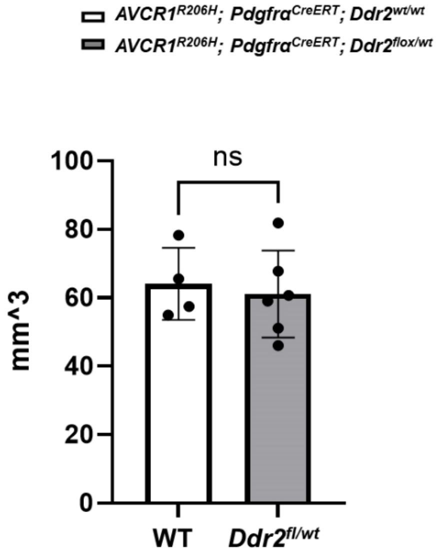

Supplementary Figure 4. Inactivation of a single Ddr2 allele does not affect ACVR1(R206H) mutant-dependent HO. *Pdgfra^{CreER};Acvr1$^{[R206H]FlEx/+}$;Ddr2$^{fl/+}$* and *Pdgfra^{CreER};Acvr1$^{[R206H]FlEx/+}$;Ddr2$^{+/+}$* mice were treated with TAM and cardiotoxin as in Fig. 6 and HO was assessed by μCT. HO volume was not significantly different between the two groups (Two-tailed T test).

Suppl. Fig5

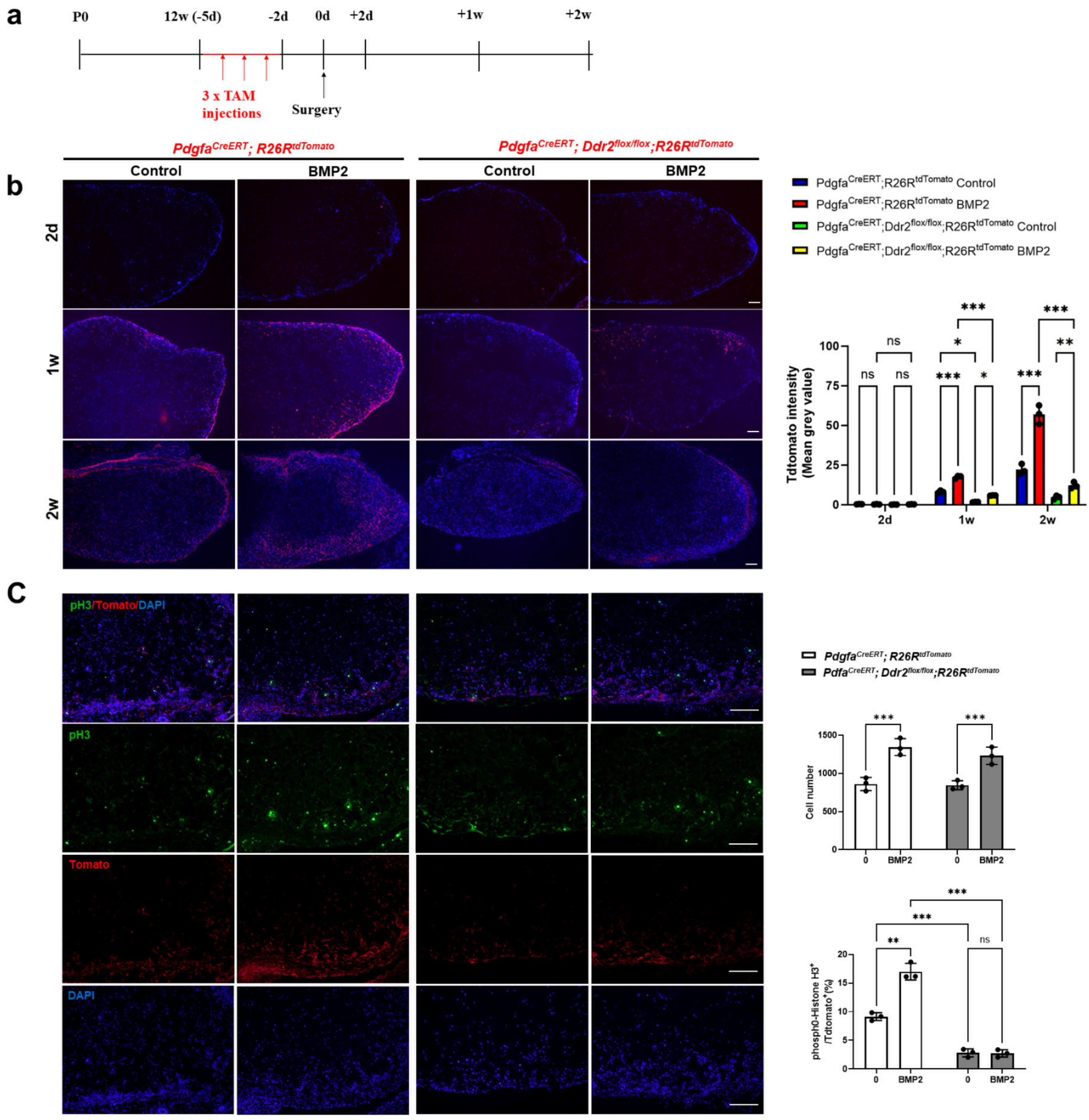

a
P0
12w (-5d)
-2d
0d
+2d
+1w
+2w
3 x TAM injections
Surgery
b
PdgfaCreERT; R26RtdTomato
PdgfaCreERT; Ddr2flox/flox;R26RtdTomato
Control
BMP2
2d
1w
2w
PdgfaCreERT;R26RtdTomato Control
PdgfaCreERT;R26RtdTomato BMP2
PdgfaCreERT;Ddr2flox/flox;R26RtdTomato Control
PdgfaCreERT;Ddr2flox/flox;R26RtdTomato BMP2
Tdtomato intensity (Mean grey value)
C
pH3/Tomato/DAPI
pH3
Tomato
DAPI
PdgfaCreERT; R26RtdTomato
PdfaCreERT; Ddr2flox/flox;R26RtdTomato
Cell number
BMP2
phosph0-Histone H3+ /Tdtomato+(%)

Supplementary Figure 5. Contribution of *$Pdgfra^{CreERT}$*-derived cells to BMP2-induced HO formation. (a) Experimental protocol. After TAM treatment, 12 week-old *$Pdgfra^{CreERT}$; $R26R^{tdTomato}$* and *$Pdgfra^{CreERT}$;$Ddr2^{fl/fl}$; $R26R^{tdTomato}$* mice received control or BMP2 implants that were harvested after 2 days, 1 week or 2 weeks for measurement of tdTomato fluorescence **(b)**. One week samples were also analyzed for total cell number (DAPI staining) and proliferation of *$Pdgfra^{CreERT}$* -derived cells using colocalization of tdTomato-labelled cells with a phospho-histone H3 (pH3) antibody **(c)**. Key: Panel b bar graph: *$Pdgfra^{CreERT}$;$R26R^{tdTomato}$* (blue bars), *$Pdgfra^{CreERT}$;$R26R^{tdTomato}$* + BMP2 (red bars), *$Pdgfra^{CreERT}$;$Ddr2^{flox/flox}$;$R26R^{tdTomato}$* (green bars), *$Pdgfra^{CreERT}$;$Ddr2^{flox/flox}$;$R26R^{tdTomato}$* + BMP2 (yellow bars). Panels c bar graphs: *$Pdgfra^{CreERT}$;$R26R^{tdTomato}$* (open bars), *$Pdgfra^{CreERT}$;$Ddr2^{flox/flox}$;$R26R^{tdTomato}$* (closed bars). Statistics: two-way ANOVA . *$P<0.05$, **$P<0.01$, ***$P<0.001$, ns not significant. Scale bar: 200μm.

Suppl. Fig6

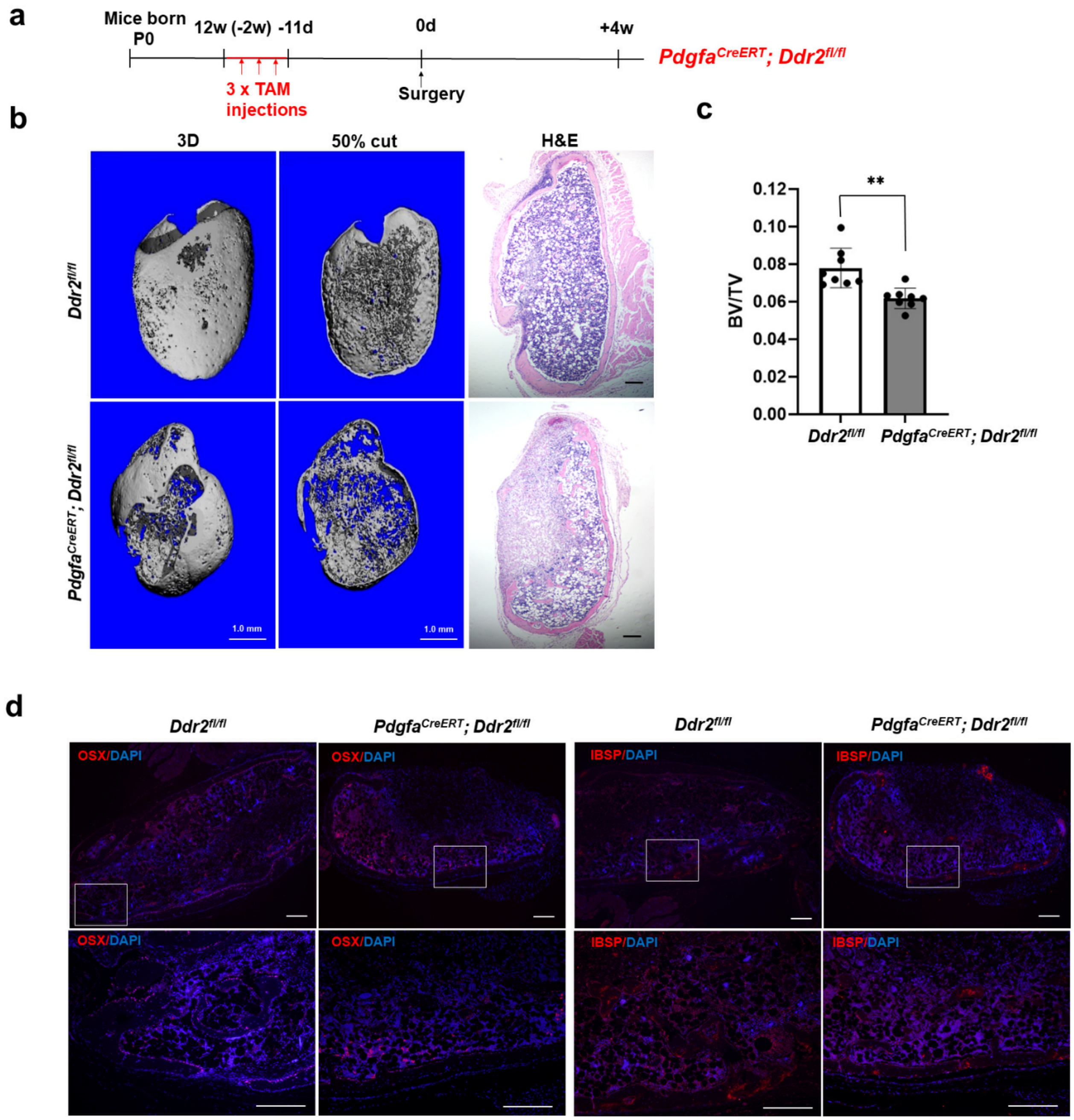

Supplementary Figure 6. Reduced sensitivity of BMP2-induced HO to inactivation of *Ddr2* in *Pdgfra*^{CreERT}-positive cells. **a)** Experimental protocol. *Ddr2*$^{fl/fl}$ and *Pdgfra*^{CreERT};*Ddr2*$^{fl/fl}$ mice were treated with TAM, BMP2 implants were placed and HO formation was measured after 4 weeks. **(b)** representative μCT images (L, center) and histology (R). **(c)** Quantification of ossicle bone volume. **(d)** OSX and IBSP IF localization. Top row, low power images; bottom row, high power image of boxed area. Scale bar: 200μm.

Suppl. Fig7

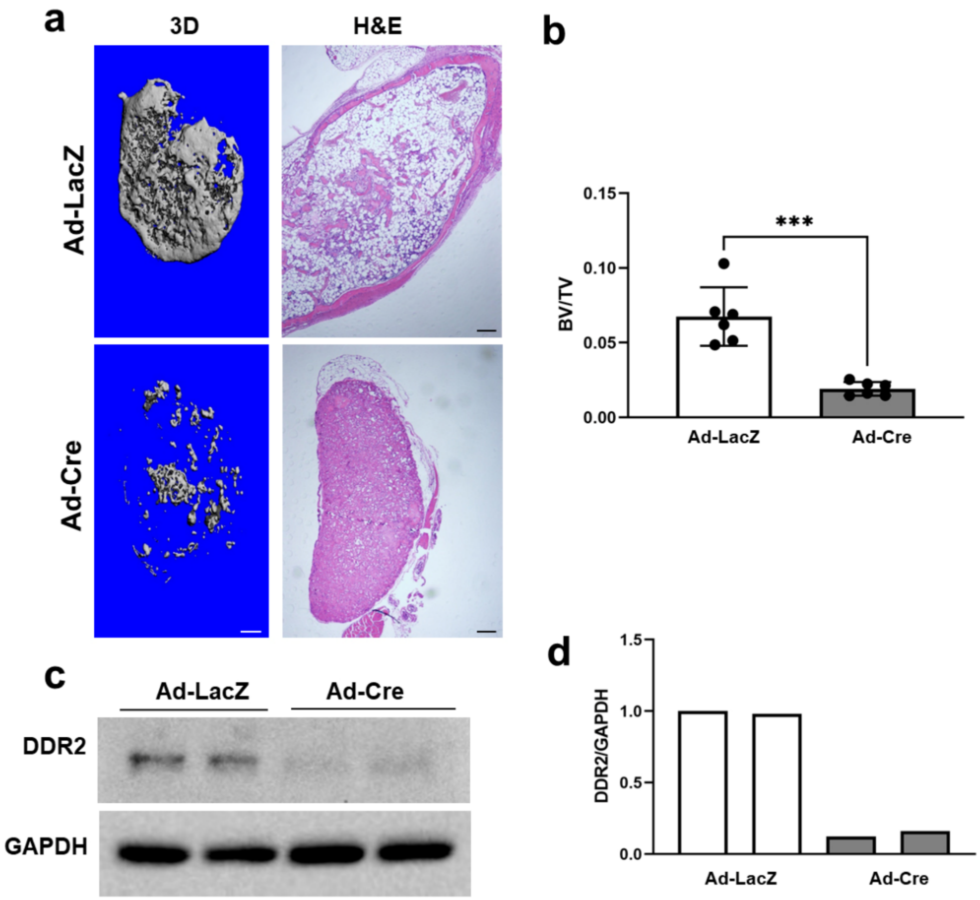

Supplementary Figure 7. Requirement for DDR2 in BMSCs-mediated bone formation. BMSCs were isolated from *Ddr2*$^{fl/fl}$ mice and treated with control (Ad-LacZ) or Cre-expressing (Ad-Cre) adenoviruses before subcutaneous implantation in immunodeficient mice as described in Methods. **(a)** Representative μCT (L) and H&E images (R) of *Ddr2*$^{fl/fl}$ derived BMSC-mediated ectopic bone formation at 4w (Adeno-LacZ versus Adeno-Cre). **(b)** Quantification of μCT results. **(c)** Western blot of DDR2 protein in BMSC cell extracts (Adeno-LacZ versus Adeno-Cre). **(d)** Quantification of DDR2 protein levels normalized to GAPDH. Unpaired t-test, ***P<0.001. Scale bar: 200μm.

Suppl. Fig8

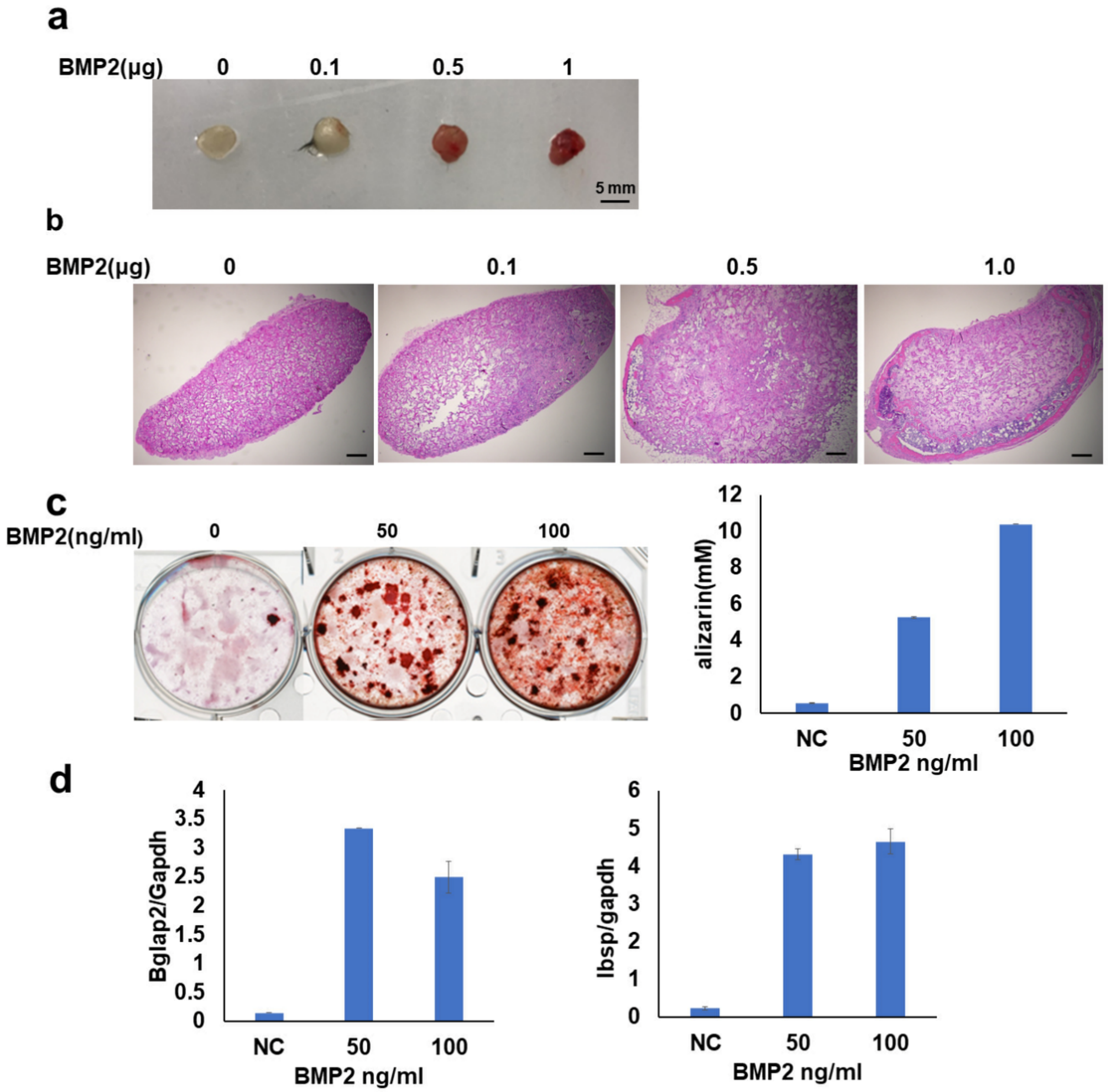

Supplementary Figure 8. In vivo and in vitro dose-dependence of BMP2-induced bone formation. (a,b) Ossicle formation in vivo. Gelatin implants were loaded with the indicated amounts of BMP2, implanted into wild type mice, and bone formation was measured after 4 weeks. Whole mounts **(a)**, histology **(b)**. **(c,d) BMP2 induction of BMSC osteoblast differentiation.** BMSCs were isolated from wild type mice and treated with the indicated concentrations of BMP2 for 3 weeks, before measurement of mineralization (Alizarin Red staining, c) or osteoblast marker mRNA (*Bglap* and *Ibsp*) induction **(d)**. scale bar(b): 200 μm
